# Glycated albumin modulates the contact system with implications for the kallikrein-kinin and intrinsic coagulation systems

**DOI:** 10.1016/j.jtha.2022.12.015

**Published:** 2022-12-27

**Authors:** Lewis J. Hardy, Dillon Bohinc, Kara L. Bane, Samantha L. Heal, Emma Hethershaw, Majid Ali, Thomas Palmer-Dench, Richard Foster, Colin Longstaff, Thomas Renné, Evi X. Stavrou, Helen Philippou

**Affiliations:** 1 Leeds Institute of Cardiovascular & Metabolic Medicine, University of Leeds, Leeds, West Yorkshire, United Kingdom; 2 Department of Medicine, Hematology and Oncology Division, CWRU School of Medicine, Cleveland, OH, USA; 3 School of Chemistry, University of Leeds, Leeds, West Yorkshire, UK; 4 Biotherapeutics Group, Haemostasis Section, National Institute for Biological Standards and Control, South Mimms, Hertfordshire, United Kingdom; 5 Institute for Clinical Chemistry and Laboratory Medicine, University Medical Centre Hamburg-Eppendorf, Hamburg, Germany; 6 Irish Centre for Vascular Biology, School of Pharmacy and Biomolecular Sciences, Royal College of Surgeons in Ireland, Dublin, Ireland; 7 Center for Thrombosis and Hemostasis, Johannes Gutenberg University Medical Center, Mainz, Germany; 8 Medicine Service, Section of Hematology-Oncology, Louis Stokes Veterans Administration Medical Center, Cleveland, Ohio, USA

**Keywords:** advanced glycation end products, albumin, diabetes mellitus, factor XII, hemostasis, inflammation, prekallikrein

## Abstract

**Background:**

Human serum albumin (HSA) is the most abundant plasma protein and is sensitive to glycation *in vivo*. The chronic hyperglycemic conditions in patients with diabetes mellitus (DM) induce a nonenzymatic Maillard reaction that denatures plasma proteins and forms advanced glycation end products (AGEs). HSA-AGE is a prevalent misfolded protein in patients with DM and is associated with factor XII activation and downstream proinflammatory kallikrein-kinin system activity without any associated procoagulant activity of the intrinsic pathway.

**Objectives:**

This study aimed to determine the relevance of HSA-AGE toward diabetic pathophysiology.

**Methods:**

The plasma obtained from patients with DM and euglycemic volunteers was probed for activation of FXII, prekallikrein (PK), and cleaved high-molecular-weight kininogen by immunoblotting. Constitutive plasma kallikrein activity was determined via chromogenic assay. Activation and kinetic modulation of FXII, PK, FXI, FIX, and FX via *in vitro*–generated HSA-AGE were explored using chromogenic assays, plasma-clotting assays, and an *in vitro* flow model using whole blood.

**Results:**

Plasma obtained from patients with DM contained increased plasma AGEs, activated FXIIa, and resultant cleaved cleaved high-molecular-weight kininogen. Elevated constitutive plasma kallikrein enzymatic activity was identified, which positively correlated with glycated hemoglobin levels, representing the first evidence of this phenomenon. HSA-AGE, generated *in vitro*, triggered FXIIa-dependent PK activation but limited the intrinsic coagulation pathway activation by inhibiting FXIa and FIXa-dependent FX activation in plasma.

**Conclusion:**

These data indicate a proinflammatory role of HSA-AGEs in the pathophysiology of DM via FXII and kallikrein-kinin system activation. A procoagulant effect of FXII activation was lost through the inhibition of FXIa and FIXa-dependent FX activation by HSA-AGEs.

## 1 | INTRODUCTION

The contact activation system (CAS) plays key roles in coagulation [1], inflammation [2], innate immunity, and fibrinolysis [3]. The CAS is activated when blood is exposed to anionic surfaces, such as glass, polyphosphate nanoparticles [4], and extracorporeal membrane oxygenation systems [5]. The CAS consists of factor (F)XII and prekallikrein (PK) with its cofactor high-molecular-weight kininogen (HK). FXII circulates in the blood as a single-chain zymogen and on binding to an anionic surface, the quiescent zymogen gains proteolytic activity (contact activation) sufficient to cleave its primary substrate PK into kallikrein (PKa) [6]. PKa may then activate non–surface bound FXII (fluid-phase activation) but this process is most efficient with the conformational rearrangements exhibited by FXII when bound to an anionic surface (solid-phase activation) [6,7]. HK is bound to PK in a noncovalent complex [8] and enhances PKa generation during contact activation, but is also a substrate for PKa. Proteolysis of HK by PKa yields cleaved HK (HKa) and the proinflammatory nonapeptide bradykinin (BK), collectively known as the kallikrein-kinin system. BK is a potent agonist of the bradykinin 2 receptor (B_2_R), which regulates cardiovascular and renal functions, such as vascular permeability, vasodilation, diuresis, and natriuresis [2].

Excessive BK generation is prevalent in patients with hereditary angioedema, a deficiency of C1 esterase inhibitors, which causes acute and paroxysmal tissue swelling [9]. Activated FXII(a) initiates the intrinsic coagulation pathway by activating FXI to FXIa, resulting in downstream thrombin generation. The sequential activation of FXIIa-initiated coagulation factors forms the basis of the activated partial thromboplastin time (aPTT) coagulation assay. FXII is an interesting coagulation factor because animal models have revealed a crucial role of FXII for thrombosis without affecting hemostasis [5,10]. This is also supported by the observation that humans with a deficiency for FXII are asymptomatic of any bleeding diathesis [11]. The primary physiologic agonist that triggers contact activation and downstream coagulation is considered to be anionic polyphosphate nanoparticles exposed on procoagulant platelets upon degranulation [4,12]. Other physiologically relevant activating anionic surfaces include mast cell heparin [7], extracellular RNA [13], neutrophil extracellular traps [14], and misfolded protein aggregates [15].

Advanced glycation end-products (AGEs) have previously been shown to trigger contact activation *in vitro*, including human serum albumin-AGE (HSA-AGE) and hemoglobin-AGE, to selectively trigger FXII-dependent PKa generation in the absence of associated intrinsic coagulation activation [15]. AGE-modification of proteins is a denaturation process that induces the loss of α-helical structures within a protein and increases the content of β-sheets and unordered structures, resulting in enhanced susceptibility of AGE-modified proteins to aggregate [16,17]. This misfolding is relevant in the study of contact activation in diabetes mellitus (DM). Persistent elevation of blood glucose levels in poorly controlled diabetic patients increases the rate of nonenzymatic glycation of proteins and results in the accumulation of AGEs in plasma and tissues [18]. HSA-AGE is found within atherosclerotic plaques [17] and has also been linked to diabetic end-organ damage including retinopathy, neuropathy, nephropathy, and coronary artery disease [19]. The mechanism underlying the reason misfolded protein aggregates selectively trigger FXII-dependent BK generation in the absence of intrinsic coagulation currently remains unsolved but higher basal levels of FXIIa, PKa, FXIa, FVIIIa, and FIXa activity have been reported in patients with DM [20–24].

In the present study, we investigated how HSA-AGE can generate FXII-dependent PKa but does not proceed to activate coagulation via the intrinsic coagulation pathway. We investigated the relationship between plasma glycation levels and contact pathway activation in diabetic patients and characterized the effects of *in vitro*–generated HSA-AGE on FXII, PK, and FXI activation and thrombus formation. Our investigations provide novel insights into the pathophysiology associated with glycation-induced protein misfolding in DM and PKa generation.

## 2 | METHODS

Lists of chemicals and reagents, criteria for human studies, blood collection, preparation of glycated albumin and surfaces for contact activation, phospholipid preparation, Thioflavin T (ThT), Congo Red (CR) assays, and circular dichroism can be found in the Supplementary Methods.

### 2.1 | Chromogenic assays

#### 2.1.1 | Continuous assays

All chromogenic assays were conducted in microtiter plate wells and absorbance was measured in a microplate reader at 405-nm wave-length. Microtiter plate wells were blocked with blocking buffer (10 mM HEPES, 150 mM NaCl, 1% PEG-20000, 0.05% Polysorbate 20 [Tween 20], pH 7.4) for 1 hour before performing chromogenic assays. Activation of FXII (100 nM) was monitored in 100 μL standard HEPES-buffered saline (HBS) buffer (10 mM HEPES, 10 μM ZnCl_2_, 150 mM NaCl, 1% PEG-8000, pH 7.4) at 37 °C with 200 μM S2302 along with reagent for aPTT termed PTT Automate (PTT-a), or HSA-AGE as agonists. PTT-a is a standard silica and phospholipid aPTT reagent, used to trigger FXII activation and the subsequent intrinsic coagulation cascade in diagnostic plasma assays. Enzymatic activity of FIXa (300 nM) or FXa (3 nM) was determined using S2765 at 3 mM or 0.7 mM, respectively. HSA-AGE was preincubated with the purified enzymes for 5 minutes before adding chromogenic substrate. Initial linear rates of reaction were measured (mODs^−1^) and expressed as % enzyme activity relative to the HBS control. The PKa-like activity was monitored in 20% (volume per volume [v/v]) sodium citrate anticoagulated platelet poor plasma in HBS buffer (10 mM HEPES, 10 μM ZnCl_2_, 50 mM NaCl, 1% PEG-8000, pH 7.4), 200 μM S2302, and agonists.

#### 2.1.2 | Discontinuous assays

FXII-dependent PKa generation was determined using 2 mM S2302. Two nanoMolar FXII was incubated with 12 nM PK ± 12 nM HK and agonists for 40 minutes before adding S2302, and 1% (v/v) PTT-a was used as a positive control. FXII-/FXIIa-dependent FXIa generation was determined by incubating 2 nM FXII with 30 nM FXI ± 30 nM HK and agonists for 3 hours, before the addition of 2 mM S2288; 1% (v/v) PTT-a or kaolin were used as positive controls. In some cases, 300 pM FXIIa was added instead of 2 nM FXII, and the incubation time was omitted. For the 2 discontinuous assays, a standard curve of PKa (0.39–30 nM) or FXIa (0.39–30 nM) activity was generated on each microtiter plate.

#### 2.1.3 | Determination of kinetic parameters

FXIa on S2288 was determined by mixing 3 nM FXIa with serial dilutions of S2288 following the addition of HSA-AGE. FIXa on FX was determined by mixing 100 nM FIXa with dilutions of FX (3.9–1000 μM), 100 μM phospholipid micelles, and 700 μM S2765 after adding HSA-AGE (0.78–50 μg.mL^−1^).

#### 2.1.4 | aPTT, prothrombin time, reptilase assays, and turbidimetric clotting assays

aPTTs, prothrombin times (PTs), and reptilase assays were performed using a STart benchtop coagulometer (Stago). For aPTTs, 47.5 μL pooled normal plasma (PNP) was added to 2.5 μL HBS or HSA-AGE. Fifty microliter PTT-a was added to the PNP mixture, then pre-warmed 50 μL 25 mM CaCl_2_ was added. For PTs, 71.25 μL PNP was added to 3.75 μL HBS or HSA-AGE before 75 μL Technoplastin-HIS were incorporated. Reptilase times were performed by mixing 2.5 μL HBS or HSA-AGE with 47.5 μL PNP. Fifty microliter Owen Koller diluent was then added and incubated for 2 minutes at 37 °C before adding 100 μL STA-Reptilase (approximately 10 U batroxobin units per mL). Some clotting assays were measured turbidometrically and prepared in the same way as aPTT assays in 96-well clear microtiter plates. Assays were performed by measuring plasma turbidity at 340 nm to monitor fibrin polymerization using a Bio Tek PowerWave HT Microplate Spectrophotometer. Clotting times were determined by measuring the time of 50% maximum clot formation.

#### 2.1.5 | Thrombus formation under flow conditions

Channel slides (Ibidi μ−Slide VI 0.1, Thistle Scientific) were half-filled with 1.3 μL 50 μg.mL^−1^ fibrillar type I collagen (Labmedics) (diluted in 500 mM acetic acid, pH 2.3) and incubated for 1 hour in a humid atmosphere at 37 °C. Slides were then blocked with an HBS-bovine serum albumin advanced (BSA) buffer (10 mM HEPES, 150 mM NaCl, 0.1% D-Glucose, 1% weight per volume) BSA, pH 7.4) for 30 minutes at 37 °C before experiments. Final concentrations of both 0.5 μg.mL^−1^ 3,3’-dihexyloxacarbocyanine iodide, [DiOC_6_(3)] (ThermoFisher Scientific) and 40 μg.mL^−1^ Alexa Fluor 647 conjugated human fibrinogen (AF647-fibrinogen) were added to freshly collected sodium citrate–anticoagulated blood samples and incubated at room temperature for 10 minutes. Two 1-mL syringes were filled with whole blood or recalcification buffer (5 mM HEPES, 110 mM NaCl, 2.7 mM KCl, 13.3 mM CaCl_2_ and 6.7 mM MgCl_2_, pH 7.4), and loaded onto an AL1800–220 Aladdin programmable 8-channel multi-syringe pump. Both syringes were connected to silicone tubing with a 0.8-mm internal diameter (Rubber BV). The 2 pieces of tubing were connected via a Y-style splitter (Thistle Scientific), which in turn connected to the channel slide via a Luer elbow connector (Thistle Scientific). Whole blood and recalcification buffer were perfused into the channel at equal flow rates, diluting whole blood by half and resulting in a final shear rate of 150s^−1^. The blood/recalcification buffer perfusion was terminated after a time allowing for sufficient thrombus formation, depending on the donor sample, usually between 20 to 40 minutes. Fluorescent images were taken using a Zeiss Axio Observer 7 microscope equipped with a Hamamatsu Flash 4 LT sCMOS camera and Definite Focus2 (Zeiss). Two-dimensional images of the total area covered by collagen for each channel were scanned at 1-minute intervals using a 10x/0.3 M27 EC Plan-Neofluar objective (Zeiss) and automated scanning stage (Zeiss). *Z*-stacks were scanned for both DiOC_6_ labeled platelets and AF-647 fibrin(ogen) using a 40×/0.95 Plan-Apochromat air objective (Zeiss). Images were processed using ZEN 3.0 with deconvolution (Zeiss).

## 3 | RESULTS

### 3.1 | In diabetic individuals, plasma FXIIa and PKa activities are constitutively elevated and correlate with poor glycemic control.

**FIGURE 1:**
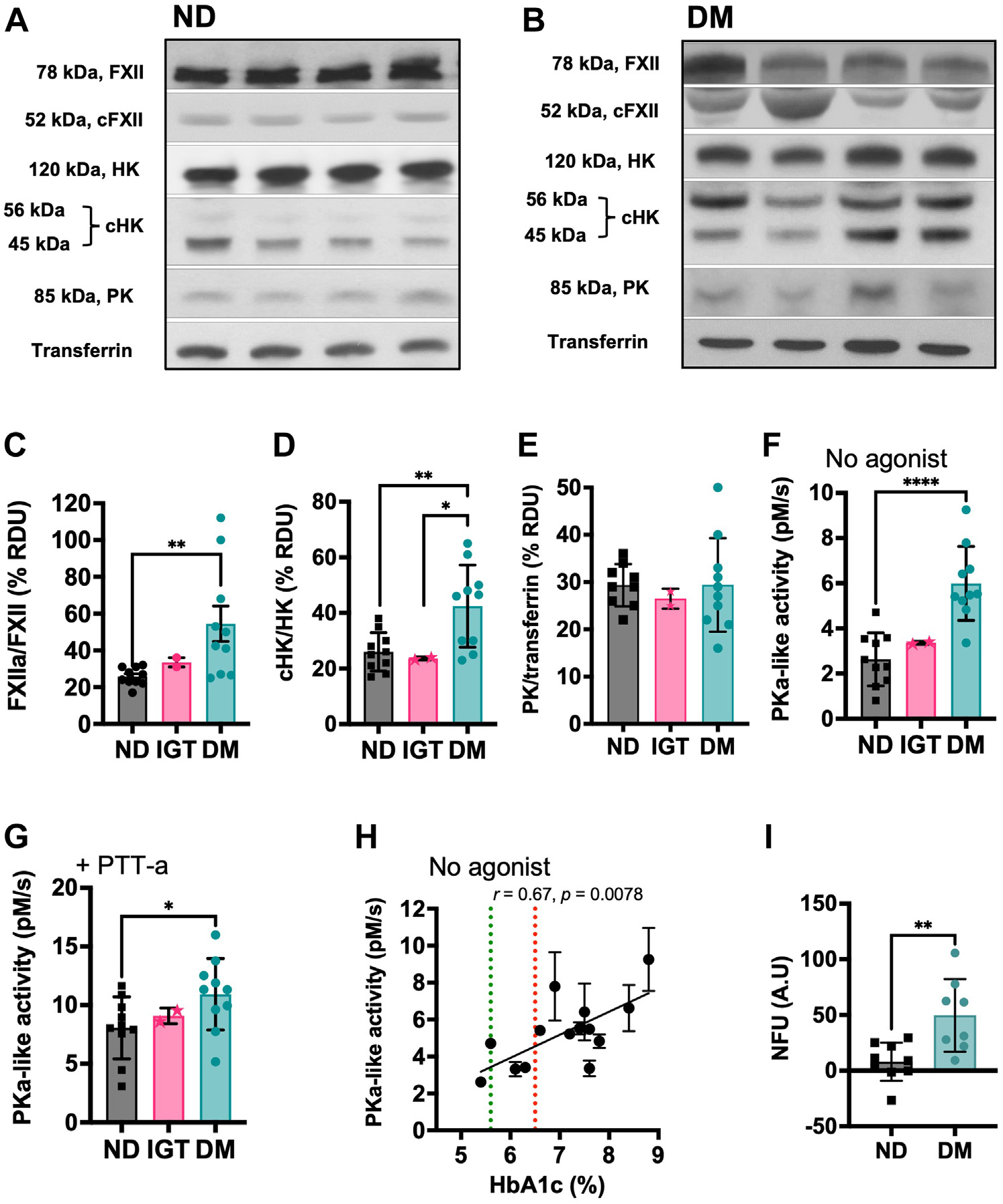
Diabetic patients have increased contact factor activity that correlates with glycated hemoglobin (HbA1c) levels. (A-B) Representative western blots of zymogen and protease fragments of FXII, intact and cHK, and prekallikrein (PK) in (A) ND and (B) DM samples. (C) Densitometry analysis of reducing western blots probing for FXII. Patients with DM (n = 10) had significantly higher cleaved FXII (representing FXIIa) levels in plasma compared with the ND group (n = 10) (*p* = .005). (D) Densitometry analysis of reducing western blots probing for HK. Patients with DM had significantly higher cHK levels than ND control (*p* = .01) and IGT groups (*p* = .03). (E) Densitometry analysis of reducing western blots probing for PK. There was no significant difference in PK antigen levels in patients with DM compared with ND and IGT samples. (F) Basal PKa-like activity in plasma was significantly higher in patients with DM compared with the ND control group (*p* = .0001) and (G) in the presence of PTT-a (*p* = .037). (H) Basal PKa-like activity in plasma positively correlated with HbA1c levels (r = 0.67, *p* = .0078). (I) DM plasma samples had significantly higher AGE-specific fluorescence compared with ND samples (*p* = .0043). Statistically significant differences were identified using Mann-Whitney nonparametric tests. cHK, cleaved high-molecular-weight kininogen; DM, diabetes mellitus; FXII, factor XII; IGT, impaired glucose tolerance; ND, nondiabetic; PKa, plasma kallikrein; RDU, relative densitometry units. Where groups are significantly different, *p < .05, **p < .001, ***p < .0005, ****p < .0001.

Plasma was obtained from 10 patients with DM (type 1 DM, n = 2; type 2 DM, n = 8), 2 individuals with impaired glucose tolerance (IGT), and 10 nondiabetic (ND) volunteers, which were matched for age and comorbidities (Supplementary Table 1). There was no difference in the PT between the groups, but patients with DM had significantly shorter aPTTs compared with the ND group (28.4 ± 3.5 seconds vs 32.1 ± 3 seconds, *p* < .05; medical center’s normal range, 26–38 seconds) (Supplementary Table 1). First, we investigated whether FXII or HK were consumed at baseline in DM compared with IGT and ND states. Plasmas were analyzed by immunoblotting for zymogen FXII, FXIIa heavy chain (cFXII), HK, cHK, and PK. Compared with the ND samples, DM plasmas exhibited a significantly higher cFXIIa ratio (50 kDa) compared with the 78 kDa FXII zymogen (representative blots; Figure 1A, B). Analysis of the 20 donor plasmas by immunoblotting and band densitometry revealed a statistically significant increase in the ratio of cFXII to FXII zymogen (*p* = .0055) in diabetic samples compared with IGT and ND samples (Figure 1C, D), reflecting FXII activation. Patients with DM had significantly higher cHK levels compared with both ND and patients with IGT (DM vs ND, *p* < .01; DM vs IGT, *p* < .05) (Figure 1A, D). In contrast, PK antigen levels were not different in samples from patients with DM patient compared with ND and individuals with IGT (Figure 1A, B, E). A chromogenic assay was then used to detect PKa-like activity in plasma, which found significantly higher levels in DM samples compared with ND, in the presence (*p* < .05) or absence of PTT-a (*p* < .01) (Figure 1F, G). The PKa-like activity was assessed using PTT-a stimulated plasmas deficient in either FVII, FIX, FX, FXI, FXII, or PK. These studies showed that S2302 proteolysis was abrogated in FXII- and PK-deficient plasmas (Supplementary Figure 1A).

The cleavage of S2302 in these experiments was attributed to PKa activity. This was because when a selective inhibitor for PKa (soybean trypsin inhibitor [SBTI]) or a selective FXIIa (corn trypsin inhibitor [CTI]) inhibitor was added to plasma before the PTT-a agonist, dose-response inhibition of both the inhibitors was observed using the S2302 substrate. However, when CTI or SBTI were added following previous incubation of PTT-a with plasma S2302 (which would generate FXIIa and PKa before the addition of the inhibitors), dose-dependent proteolysis was inhibited by CTI but not SBTI, which demonstrates that PKa rather than FXIIa was responsible for S2302 proteolysis (Supplementary Figure 1B). Constitutive PKa-like activity in plasma, in the absence of PTT-a, positively correlated with glycated hemoglobin levels (r = 0.67, *p* < .01) (Figure 1H). A fluorescent assay was used to detect AGE-peptides in plasma as previously described [25] and showed that plasmas of patients with DM had significantly higher AGE-specific fluorescence compared with the ND samples (49.7 ± 11.53 A.U vs 7.95 ± 5.71 A.U; *p* < .01, Figure 1I).

### 3.2 | HSA-AGE induces FXII-dependent PKa generation

**FIGURE 2:**
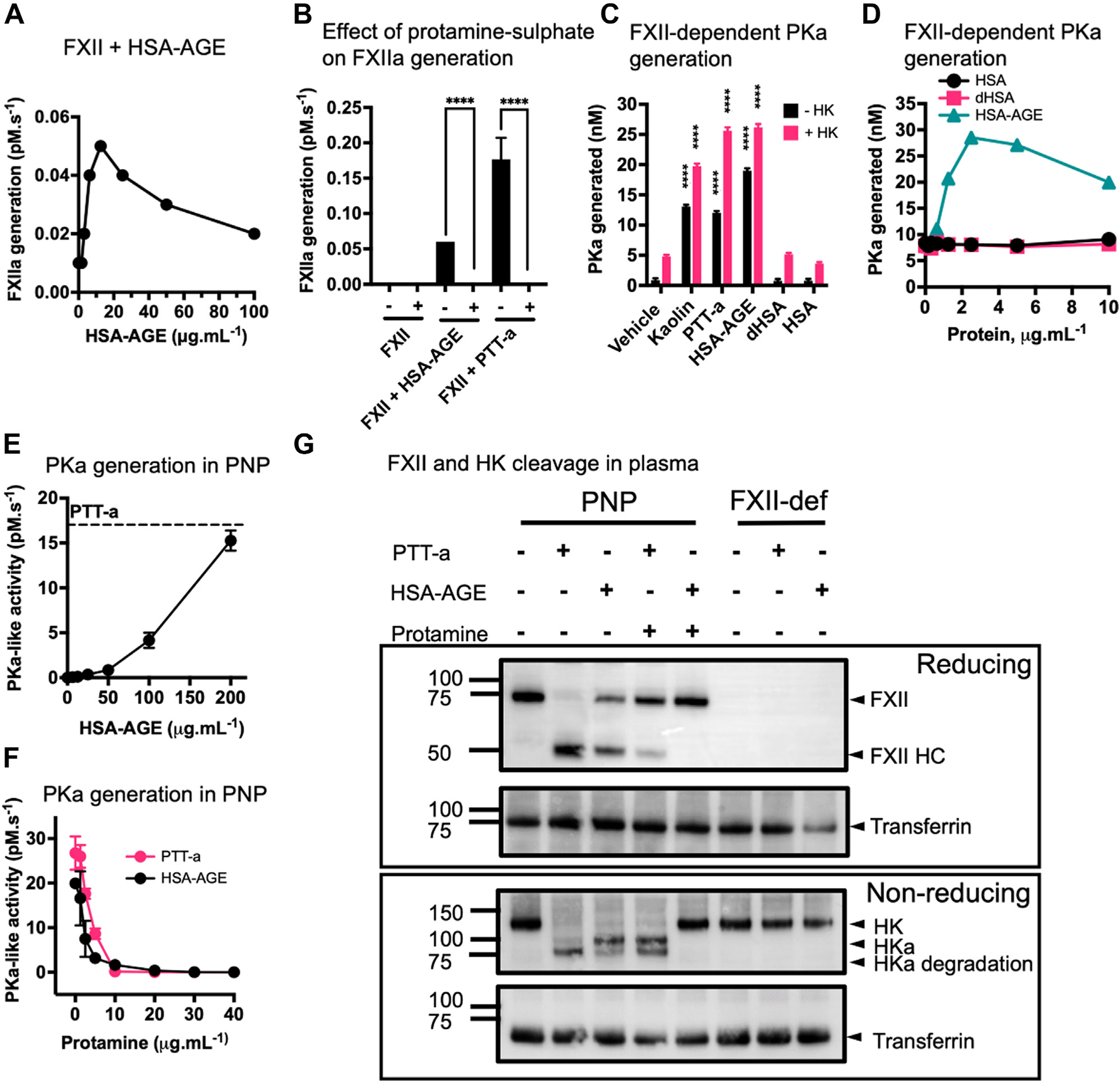
HSA-AGE induces FXII-dependent Pka generation in purified reactions and plasma. (A) FXII autoactivation was induced by incubating purified FXII with HSA-AGE (1.56–100 μg.mL^−1^). (B) Protamine sulfate (+) inhibits FXII autoactivation by PTT Automate (PTT-a) (1% v/v) or HSA-AGE (2.5 μg.mL^−1^). HEPES-buffered saline (HBS) vehicle depicted as (−), ****(*p* < .0001), where groups are significantly different in the absence of HK to the control, ****(*p* < .0001), where groups are significantly different from HK to the control. (C) Purified FXII-dependent PKa generation. Kaolin (50 μg.mL^−1^), PTT-a (1% v/v), HSA-AGE, dHSA, and native HSA (all 2.5 μg.mL^−1^) were incubated with FXII and PK for 40 minutes before adding S2302. Reactions were performed with HK (pink bars) or without (black bars) HK. PKa generation was quantified by interpolation of the rates of S2302 cleavage against a standard curve of PKa activity, ****(*p* < .0001), where groups are significantly different to the relative control without the presence of anionic surfaces. (D) Purified FXII-dependent PKa generation by HSA-AGE (1.56–10 μg.mL^−1^). PKa generation was absent when the same concentration ranges of native HSA or dHSA were used. (E) Rates of PKa-like activity in plasma. S2302 cleavage was monitored in the presence of serial dilutions of HSA-AGE (3.125–200 μg.mL^−1^) and PTT-a (5% v/v) reagent (top dashed line). (F) Dose-dependent inhibition of PKa-like activity in plasma triggered by HSA-AGE (200 μg.mL^−1^), PTT-a (5% v/v), and protamine sulfate (1.58–40 μg.mL^−1^). (G) Samples containing HSA-AGE (200 μg.mL^−1^) or PTT-a (5% v/v) were incubated for 3 hours with PNP or FXII-deficient plasma. Protamine sulfate addition is indicated as appropriate. Samples were separated via Sodium Dodecyl Sulfate-Polyacrylamide Gel Electrophoresis (SDS-PAGE) followed by immunoblotting. Membranes were probed with polyclonal anti-human FXII or polyclonal anti-human HK antibodies. AGE, advanced glycation end products; dHSA, heat denatured; FXII, factor XII; HSA, human serum albumin; HK, high-molecular-weight kininogen; IGT, impaired glucose tolerance; ND, nondiabetic; PK, prekallikrein; PKa, plasma kallikrein.

As a study prerequisite, we first prepared BSA-AGE to investigate the effect of AGE-modified proteins on contact activation (Supplementary Figures 2 and 3), which then provoked the rationale to use human-derived protein. The prolonged HSA incubation with glucose-6-phosphate–generated HSA-AGE that induced amidolytic activity of purified FXII toward S2302 (autoactivation) (Figure 2A). FXII autoactivation by HSA-AGE or PTT-a was absent following the addition of the cationic peptide protamine sulfate (Figure 2B). A discontinuous FXII-dependent PKa generation chromogenic assay was subsequently performed. Robust PKa generation was triggered by 2 nM FXII incubated with 12 PK ± 12 nM HK, 1% PTT-a (v/v), 50 μg.mL^−1^ kaolin, or 2.5 μg.mL^−1^ HSA-AGE (Figure 2C). Native HSA or heat-denatured HSA (dHSA) (both 2.5 μg.mL^−1^) failed to trigger FXII-dependent PKa generation (Figure 2D) (effects of glycation and heat denaturation on albumin are shown in Supplementary Figure 2E, F). The presence of HK in these experiments significantly enhanced the FXII-dependent PKa generation with each activating surface (Figure 2C), and FXII omission in these assays failed to generate any PKa or FXIa (see below) (Supplementary Figures 4 and 5). Moreover, ThT and CR binding assays were performed on HSA samples. Spectral ThT changes were observed in samples containing dHSA and HSA-AGE compared with native HSA, which is indicative of protein misfolding and the formation of amyloid structures (Supplementary Figure 6A). However, the CR assay showed an increase in spectral CR changes only with dHSA (Supplementary Figure 6B).

### 3.3 | HSA-AGE generates PKa in plasma

HSA-AGE was added to PNP and dose-dependently triggered PKa-like activity (Figure 2E). Protamine sulfate dose-dependently inhibited the PKa-like activity in PNP triggered by either 200 μg.mL^−1^ HSA-AGE or 1% (v/v) PTT-a (Figure 2F). PNP or FXII-deficient plasma was then mixed with 200 μg.mL^−1^ HSA-AGE or 1% (v/v) PTT-a ± 40 μg.mL^−1^ protamine sulfate and analyzed by immunoblotting (Figure 2G). Moreover, HSA-AGE and PTT-a both induced FXII activation, indicated by a reduction of FXII band intensity (80 kDa) and the appearance of the FXII heavy chain (50 kDa). Parallel to FXII activation, HSA-AGE caused HK cleavage seen by the loss of HK band intensity (120 kDa) and the appearance of HK cleavage products. Protamine sulfate inhibited reactions containing either agonist in PNP. Hence, FXII-deficient plasma could not support HK degradation triggered by PTT-a or HSA-AGE.

### 3.4 | HSA-AGE is an allosteric inhibitor of FXIa in purified reactions and does not have procoagulant properties in plasma

**FIGURE 3:**
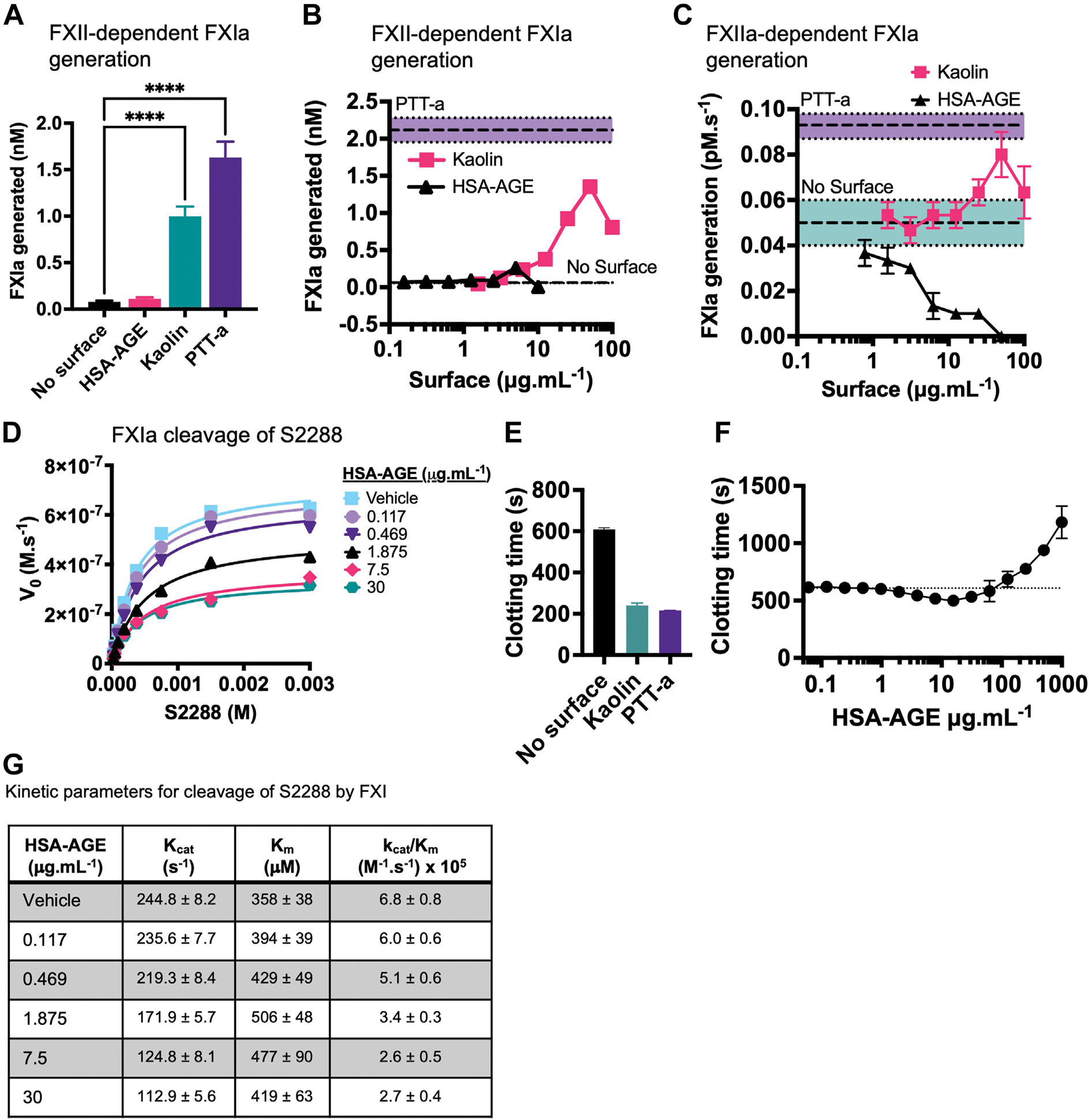
HSA-AGE inhibits FXIa in purified conditions and has weak procoagulant properties in plasma. (A) Purified FXII-dependent FXIa generation assays were performed by incubating FXII, FXI, and HK in the absence of a surface, kaolin, PTT Automate (PTT-a), or HSA-AGE for 3 hours before the chromogenic substrate S2288 was added. (B) Purified FXII-dependent FXIa generation assays were then performed with serial dilutions of kaolin or HSA-AGE. FXIa generated with PTT-a control or no surface is shown as the top and bottom dashed lines, respectively. (C) Purified FXIIa-dependent FXIa generation assays were then performed with serial dilutions of kaolin or HSA-AGE and rates of FXIa generation (pM.s^−1^) with no surface or PTT-a is shown as upper and lower dashed lines, respectively. Colored bands surrounding FXIa generation with no surface or PTT-a represent the SD of the mean. (D) Kinetic analysis of purified FXIa cleavage of S2288 in the presence of HSA-AGE. FXIa was incubated with a 2-fold dilution series of S2288 (0.011 × 10^−3^ to 3 mM) and HSA-AGE (0.117–30 μg.mL^−1^). Data points represent mean ± SD. (E) Turbidimetric plasma clotting assays were performed using pooled normal plasma with kaolin (0.12–1000μg.mL^−1^) or HSA-AGE. The time to 50% clotting was then measured for control conditions containing kaolin, PTT-a, and no surface. (F) Turbidimetric plasma clotting assays with a titration of HSA-AGE were obtained, and baseline readings obtained with no surface are shown as the dashed line. Points represent mean ± SEM, n = 3 independent experiments. (G) Kinetic parameters obtained from (D). Data are presented as mean ± SD. AGE, advanced glycation end products; dHSA, heat denatured HSA; FXII, factor XII; HSA, human serum albumin; HK, high-molecular-weight kininogen.

FXII-dependent FXIa generation chromogenic assays were performed by incubating 2 nM FXII, 30nM FXI ± 30 nM HK with either 1% PTT-a (v/v), 50 μg.mL^−1^ kaolin, or 2.5 μg.mL^−1^ HSA-AGE (Figure 3A). FXIa was generated by PTT-a or kaolin but was almost absent when using the same HSA-AGE concentration (2.5 μg.mL^−1^) used to trigger optimal FXII-dependent PKa generation (Figure 2D). HSA-AGE (0.156–10 μg.mL^−1^) or kaolin (1.56–100 μg.mL^−1^) was then titrated into the FXII-dependent FXIa generation assay. Although kaolin dose-dependently increased FXIa generation with maximal FXIa generation reaching 1.40 ± 0.09 nM at 50 μg.mL^−1^, HSA-AGE generated a maximum of 0.263 ± 0.019 nM using 5 μg.mL^−1^ (Figure 3B). These assays were then performed with 300 pM full-length alpha (α)FXIIa rather than 2 nM FXII zymogen to ensure that the differences observed in Figure 3B were not due to an FXII activation delay (efficient FXII activation occurs as a result of a positive feedback loop requiring PKa which was absent in these reactions) [26]. In the absence of a surface, FXIIa triggered 0.05 ± 0.01 pM.s^−1^ FXIa generation, which increased after the addition of kaolin (1.562–100 μg.mL^−1^) but was dose-dependently inhibited by HSA-AGE (0.78–50 μg.mL^−1^) (Figure 3C).

The kinetics of FXIa toward S2288 with HSA-AGE (Figure 3D) was studied. To assess the procoagulant (or lack of) potential of HSA-AGE in plasma, a turbidimetric plasma-clotting assay was performed and the time to 50% clot formation was measured. Control conditions containing plasma, CaCl_2_, a phospholipid mixture of 20% phosphatidylserine:20% phosphatidylethanolamine:60% phosphatidylcholine to mimic an activated platelet surface, and either no surface (HBS vehicle), PTT-a, or kaolin gave clotting times of 608.9 ± 8.3 seconds, 212.0 ± 4.0 seconds, and 240 ± 6.9 seconds, respectively (Figure 3E). HSA-AGE titrations (0.06–1000 μg.mL^−1^) were added to plasma in procoagulant conditions; clotting times began to shorten from baseline readings (no surface, 608.9 ± 8.3 seconds), with the shortest clotting time of 499.7 ± 6.5 seconds noted with the 15.25 μg.mL^−1^ HSA-AGE concentration but began to get prolonged beyond baseline readings (Figure 3F). A summary of the effect of HSA-AGE on the kinetics of FXIa toward S2288 from Figure 3D, is shown in Figure 3G, which led to dose-dependent decreases in the catalytic constant (k_cat_) of FXIa with minimal effects on the Michaelis constant (K_m_) this is a characteristic of noncompetitive allosteric enzyme inhibition.

### 3.5 | Intrinsic pathway coagulation is inhibited by HSA-AGE

**FIGURE 4:**
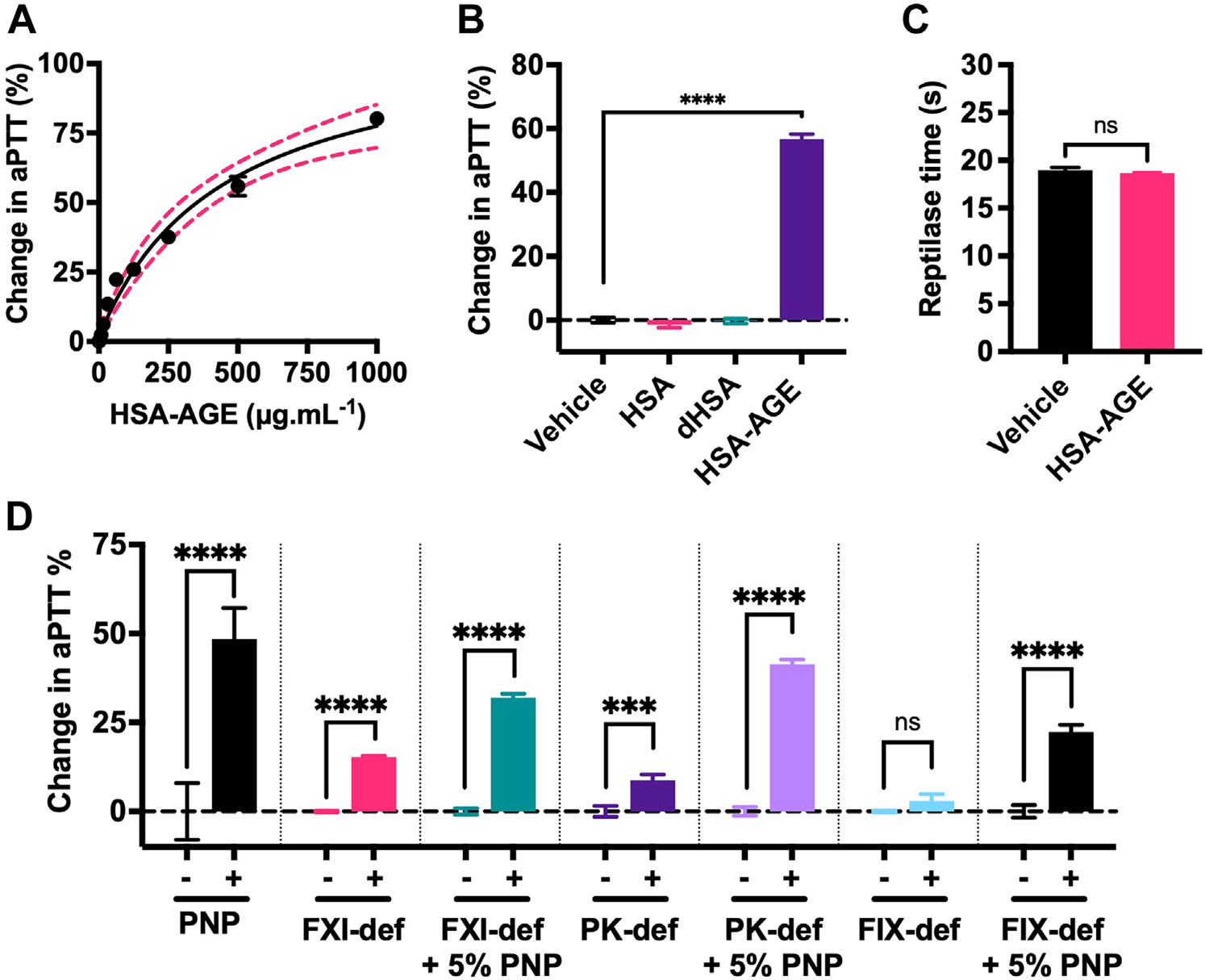
HSA-AGE has anticoagulant effects in plasma. (A) Dose-dependent prolongation of the aPTT with HSA-AGE (0.97–1000 μg.mL^−1^) using PNP. Data expressed as % mean change ± SD relative to control (aPTT time of PNP with HEPES-buffered saline [HBS] vehicle). (B) aPTT of PNP with the vehicle, HSA, dHSA, or HSA-AGE (all 400 μg.mL^−1^). Data are expressed as % mean change ± SD relative to the presence of PTT Automate (PTT-a) only (labeled as the vehicle). (C) PNP reptilase time in the presence of an HBS vehicle or HSA-AGE (400 μg.mL^−1^). (D) Plasma mixing experiments. aPTTs of PNP or plasma with deficiencies of FXI, PK, or factor (F)IX. aPTT assays of deficient plasmas were also incubated separately after adding 5% PNP. aPTT assays were performed in the presence of HBS vehicle (−) or HSA-AGE (400 μg.mL^−1^) (+). Data are presented as % mean change ± 95% CI relative to the appropriate control plasma aPTT. One-way analysis of variance was performed with Sidak multiple comparisons test. AGE, advanced glycation end products; aPTT, activated partial thromboplastin time; dHSA, heat denatured HSA; FXI, factor XI; HSA, human serum albumin; NS, no statistical difference; PK, prekallikrein, PNP, pooled normal plasma. Where groups are significantly different, ***p < .0005, ****p < .0001.

aPTT assays were performed with HSA-AGE using PNP to investigate any anticoagulant effects. HSA-AGE dose-dependently prolonged the aPTT and 400 μg.mL^−1^ HSA-AGE prolonged the aPTT by 50% (31.6s to 47.4; Figure 4A). Equal concentrations of HSA or dHSA (400 μg.mL^−1^) showed no effect on the aPTT (Figure 4B). HSA-AGE (400 μg.mL^−1^) did not affect the PNP reptilase time, suggesting fibrinopeptide-A cleavage or fibrin oligomerization is unaltered (Figure 4C). aPTT-triggered plasma mixing experiment results were then conducted to elucidate which intrinsic coagulation factor(s) were inhibited by HSA-AGE (Figure 4D). HSA-AGE (400 μg.mL^−1^) prolonged the aPTT of PNP, FXI-, and PK-but not FIX-deficient plasma. These data suggest that HSA-AGE inhibits the function or activation of FIXa. Reconstituting FIX-deficient plasma with 5% PNP, restored the inhibitory effect of HSA-AGE on aPTT assays. Determinations of aPTTs (in the absence of other reagents) for PNP, FXII-, PK-, FXI-, and FIX-deficient plasmas are shown in Supplementary Figure 7.

### 3.6 | HSA-AGE is a competitive inhibitor of FIXa-mediated FX activation

**FIGURE 5:**
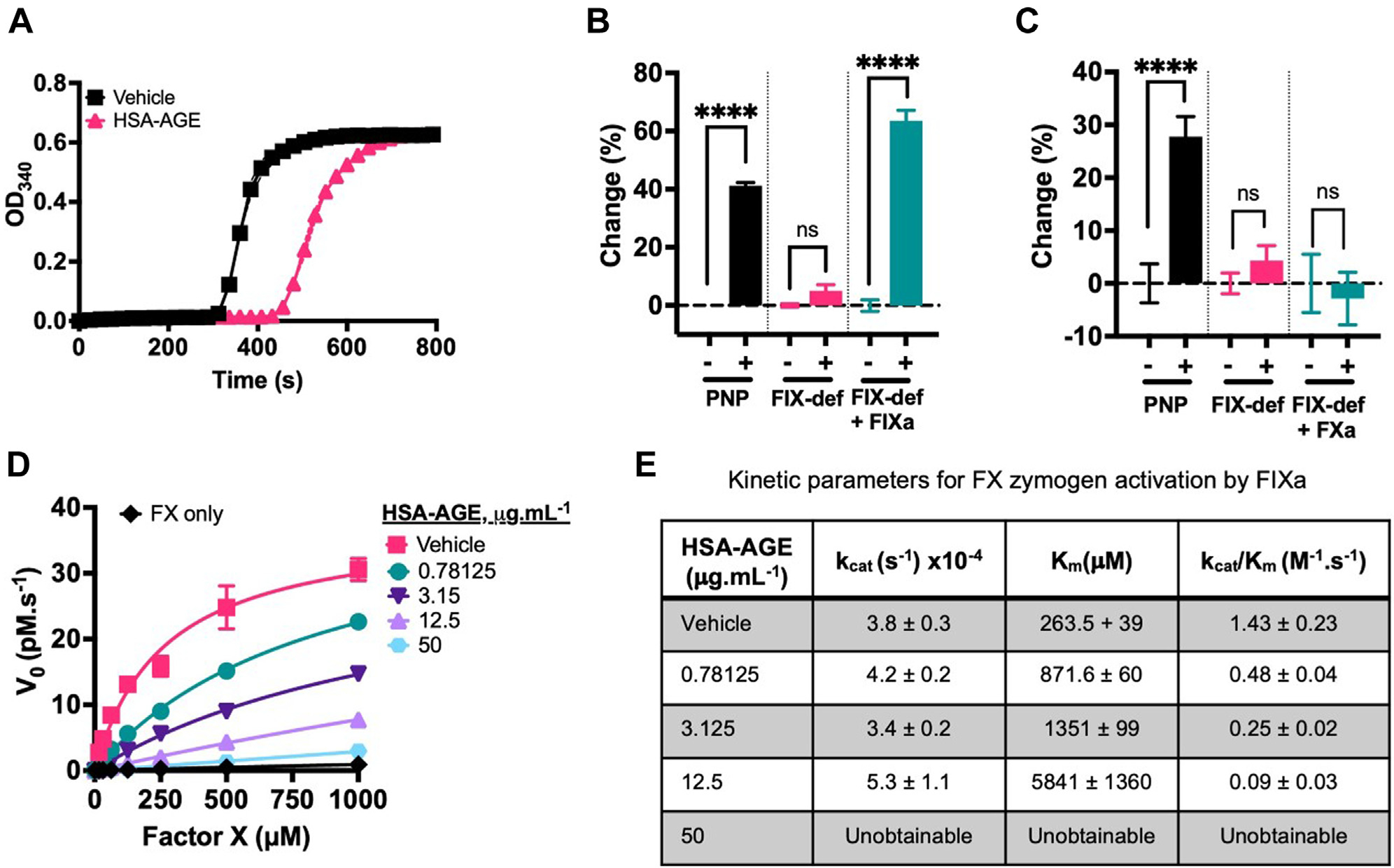
HSA-AGE inhibits activated FIX (FIXa)-dependent FX activation. (A) Clot formation spectra of PNP initiated by PTT Automate (PTT-a) with HBS vehicle or HSA-AGE (400 μg.mL^−1^). (B) Clotting of PNP, FIX-deficient plasma or FIXa-deficient plasma with FIXa addition (948 pM) was initiated by CaCl_2_ and PTT-a with HBS vehicle (−) or HSA-AGE (400 μg.mL^−1^) (+). Data presented as % mean change ± SD of time to clot relative to the appropriate control plasma. (C) A similar experiment as in (B) was performed; however, FXa (102 pM) was added to FIX-deficient plasma with HBS vehicle or HSA-AGE. (D) Kinetic analysis of FIXa activation of FX in the presence of HSA-AGE. Reactions of FIXa-dependent FX activation were performed in the presence of HSA-AGE (0.78–50 μg.mL^−1^). (E) Table of kinetic parameters determined from (E) for FIXa-dependent FX activation. K_m_ and k_cat_ values are presented as mean ± SD. AGE, advanced glycation end products; FX, factor X; HSA, human serum albumin; NS, no statistical difference; OD, optical density; PNP, pooled normal plasma. Where groups are significantly different, ****p < .0001.

A PTT-a–triggered turbidimetric clotting assay was then used to study the effects of HSA-AGE on fibrin formation in plasma (Figure 5A). Clotting times from absorbance spectra initiated by PTT-a were measured using FIX-deficient plasma, which again was unaffected by the presence of HSA-AGE. The addition of 948 pM FIXa to PTT-a stimulated FIX-deficient plasma restored the clotting time to the normal range and HSA-AGE prolonged the clotting time in these conditions (Figure 5B). In a separate assay, adding 102 pM FXa to PTT-a stimulated FIX-deficient plasma did not affect the clotting time (Figure 5C). Taken together, these data suggest HSA-AGE inhibits FX activation by FIXa in plasma. In purified reactions consisting of either 300 nM FIXa or 3 nM FXa, HSA-AGE did not affect the rate of proteolysis of S2765 by either enzyme (Supplementary Figure 8). We subsequently investigated purified FIX-dependent FX activation in a purified system (where cofactors FVIIIa and FVa were absent) with a chromogenic assay. In a FIXa-dependent FX activation assay, FX activation was inhibited in a dose-dependent fashion by HSA-AGE (Figure 5D). However, the plasma experiments shown in Figure 4 were performed in plasma that contained all relevant cofactors, further supporting the hypothesis that HSA-AGE directly inhibited FIXa-dependent FX activation. FIXa cleaved FX with a K_m_ 263.5 ± 39 μM and k_cat_ 3.8 ± 0.3 (×10^−4^) s^−1^ and HSA-AGE increased the K_m_ of FIXa-mediated FX activation with minimal effects toward the k_cat_, indicative of competitive inhibition (Figure 5D, E).

### 3.7 | HSA-AGE inhibits collagen-dependent fibrin formation in whole blood at a venous shear rate

**FIGURE 6:**
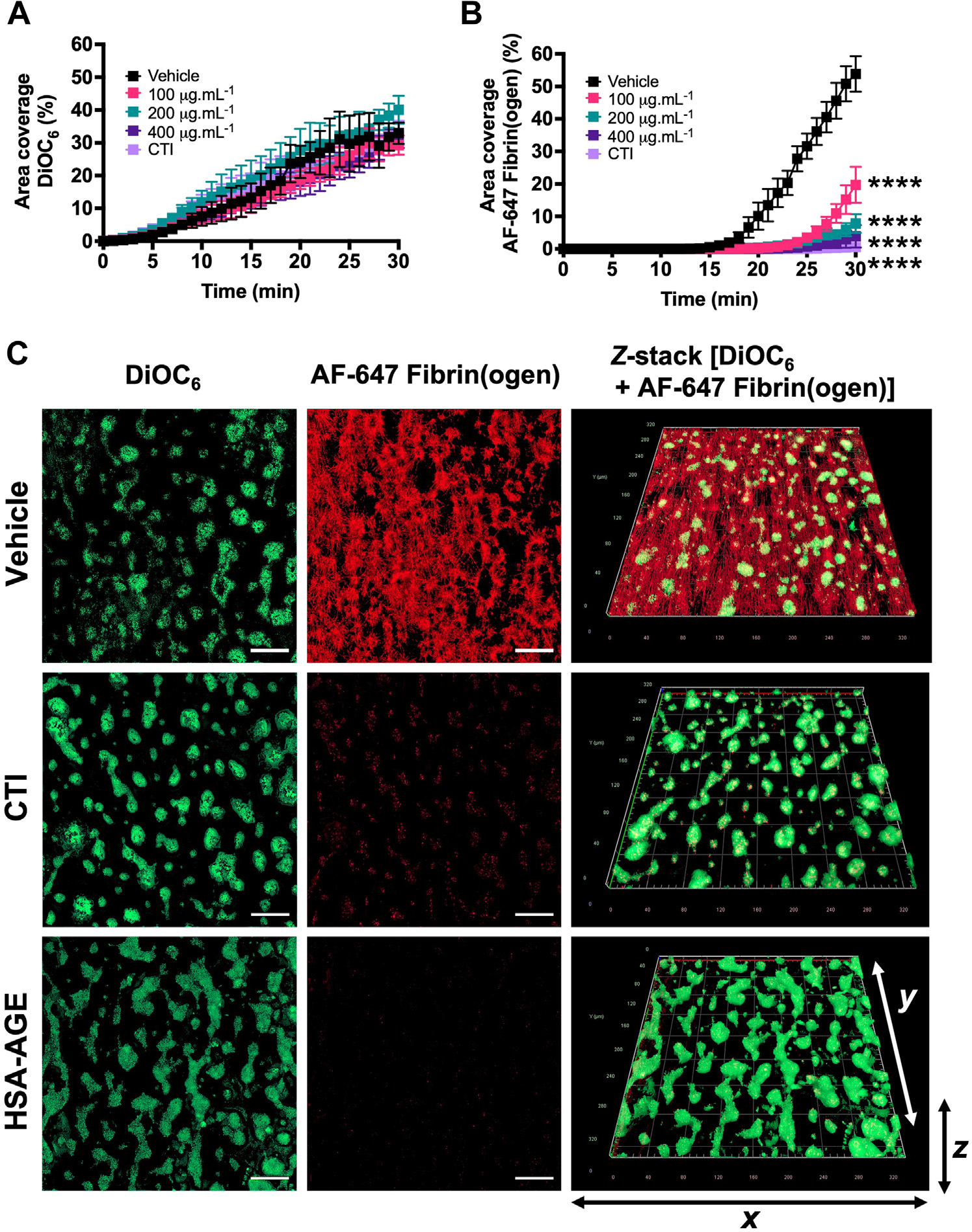
Collagen-dependent thrombus formation under venous flow conditions in human blood is inhibited by HSA-AGE (A) DiOC_6_ (platelets) and (B) AF-647 Fibrin(ogen) area coverage determined from thrombi formed from blood pretreated with HEPES-buffered saline (HBS) or HSA-AGE (100–400 μg.mL^−1^) for 5 minutes before perfusion. CTI (1.6 μM) was added as an internal control to show an FXII dependency of fibrin(ogen) accumulation. Curves are constructed as mean ± SEM of 4 donors. (C) Representative images of thrombi formed in the presence of HBS vehicle, CTI, or HSA-AGE (400 μg.mL^−1^) and labelled with DiOC_6_ (green) indicative of platelets and fibrin with AF-647 fibrin(ogen) (red). Scale bars, 50 μm. AGE, advanced glycation end products; CTI, corn trypsin inhibitor; FXII, factor XII; HSA, human serum albumin; NS, no statistical. Where groups are significantly different from the vehicle control, ****p < .0001.

Flow experiments were performed at a shear rate of 150s^−1^, as a representative of venous flow conditions, to investigate HSA-AGE effects on collagen-dependent platelet-mediated intrinsic coagulation. To show the model is dependent on FXIIa, we added 1.6 μM CTI, which blocked FXIIa-driven fibrin formation but did not affect the platelet surface area coverage. We then added HSA-AGE to whole blood, which dose-dependently blocked FXII-driven fibrin formation but did not affect the platelet surface area coverage. These data show that HSA-AGE inhibits FXII-driven coagulation using physiologic agonists in whole blood and flow conditions (Figure 6).

## 4 | DISCUSSION

A key objective of this study was to determine how misfolded protein aggregates can trigger contact activation without associated intrinsic coagulation. We chose to investigate this phenomenon using *in vitro*–generated HSA-AGE because of the physiologic relevance associated with DM. Our findings suggest that HSA-AGE activates FXII-dependent PKa generation and simultaneously inhibits procoagulant responses through selective interference of FXIa- and FIXa-dependent FXa generation. We have established that although HSA-AGE activates FXII, the resulting overall effect is more proinflammatory (via kallikrein-kinin system activation), rather than procoagulant because of the inhibitory effect of HSA-AGE at various points of the coagulation cascade. Interestingly, HSA-AGE appears to be a physiologic equivalent to the *in vitro* effect of the synthetic polyanion dextran sulfate, which also triggers FXII and PK generation but inhibits thrombin generation [27].

We first investigated the activity of CAS in diabetes. The DM group in our study had a mean glycated hemoglobin level of 7.58% (≥6.5% threshold for DM). The results show that DM is associated with elevated levels of FXIIa and cHK antigen, which we determined by immunoblotting. Plasma of patients with DM also showed significantly higher constitutive PKa activity via a chromogenic approach when compared with ND controls, which positively correlated with glycated hemoglobin %. The AGE-specific fluorescence emitted by plasma of patients with DM was approximately 6.22-fold higher than ND controls, which agrees with previous findings [28–30]. Previous studies have shown that high PKa activity in diabetics is associated with hypertension, proteinuria, nephropathy, increased vascular permeability, and macular edema [20,31–34]. The constitutive PKa activity seen in diabetic patients’ plasma is an enigma, but we speculate that AGE-proteins may contribute to this elevated basal PKa activity given that HSA-AGE alone is a potent agonist of the CAS. Further studies are required to delineate the activation mechanism(s) of PKa in patients with DM using *in vivo*–acquired material, which was beyond the scope of this study.

The current study’s data agree with previous findings that patients with DM have slightly shorter aPTTs but not PTs, compared with euglycemic controls [24], although these remained within the institutional normal range. Prior data suggest PKa contributes to these shorter aPTT times and the general prothrombotic state of patients with DM [20,21,24,35]. Our study shows that although aPTTs were indeed shorter in DM subjects, when HSA-AGE is added to normal plasma, it prolongs aPTT. This indicates that patients with DM have other factors beyond the anticoagulant effect of HSA-AGE contributing toward a prothrombotic phenotype, such as elevated FVIII levels [23]. Glycation of other proteins, such as fibrinogen and plasminogen, has been shown to contribute to the prothrombotic state of patients with DM [36,37]. However, hyperglycemia is not the only metabolic dysfunction that contributes toward protein misfolding in DM, the disease is also associated with a complicated milieu of metabolic imbalances including oxidative and carbonyl stress [36,38], which also modifies protein structures *in vivo* [39].

Our studies with HSA-AGE agree with previous findings by Maas et al. [15], who described that amorphous protein aggregates trigger FXII-dependent PKa generation without an associated FXIa generation. Here we show that HSA-AGE triggers FXII-dependent PKa generation with a proportional anticoagulant response and report for the first time, to our knowledge, that HSA-AGE inhibits FXIa and separately FIXa-dependent FXa generation; therefore, accounting for the observed anticoagulant effect. Our investigation focused on characterizing *in vitro*–generated HSA-AGE added to PNP, which is devoid of the complex metabolic milieu or other glycated plasma proteins. Future studies will be required using advanced purification methods to obtain AGE-modified proteins from the plasma of patients with DM to fully delineate the effects of isolated AGE-modified proteins toward contact activation and intrinsic coagulation.

AGEs are generated *in vitro* by long-term incubation with glucose, which is characterized by autofluorescence, inter- and intramolecular crosslinking to form both soluble and nonsoluble amorphous, amyloid-like anionic high-molecular-weight aggregates with decreased proteolytic susceptibility and protein clearance *in vivo* [16,19,40]. Ikeda et al. [40] previously developed an anti-AGE monoclonal antibody, 6D12, raised by immunizing mice with *in vitro* preparations of BSA-AGE. Experiments using 6D12 found common epitopes are shared with other AGE-modified proteins and peptides [40]. Immunochemical studies using the 6D12 antibody recognized epitopes on histology sections obtained from patients with diabetic nephropathy and neuropathy, atherosclerosis, and Alzheimer disease [41–44]. This supports that epitopes formed during *in vitro* AGE formation have physiologic relevance. The epitope shared among AGE-proteins was later found to be *N*ε-(carboxymethyl)lysine, which is a modified lysine adduct formed on the protein surface in the advanced stages of the Maillard reaction, which increases the net negative charge of AGE-proteins [40]. HSA-AGE has a similar structure to toxic amyloid oligomers and has been identified in pathologies such as atherosclerosis, renal failure, and Alzheimer and Parkinson diseases [15–17,45].

Our results showed HSA-AGE triggered purified FXII autoactivation and FXII-dependent PKa activity in both purified and plasma-based assays. The anionic charge dependency of these reactions was seen following the addition of the cationic peptide protamine sulfate to reactions triggered by HSA-AGE. Heat denaturation of albumin is a simple method used to induce fibril formation in albumin rich in α-helices, as opposed to the high β-sheet content of HSA-AGE [46]. ThT and CR are diagnostic fluorescent dyes that undergo spectral changes in fluorescence once bound to misfolded protein and amyloid-like structures; historically, these dyes have been used to detect amyloid plaques in patient histology samples. However, dHSA showed strong binding to ThT and CR but did not trigger FXII-dependent PKa generation, indicating that ThT or CR binding to misfolded proteins does not always translate to an ability to activate FXII. Although HSA-AGE resulted in the FXIIa and PKa regeneration, it did not trigger FXII-dependent FXIa generation but directly inhibited FXIa activity in purified assays. This is interesting because FXIa can also undergo allosteric inhibition by polyanions [47,48]. Further analysis studying how misfolded proteins interact with the FXI apple (A1–4) or catalytic domains to allosterically cause dysfunction of the catalytic domain is warranted in further studies. One possible mechanism may be related to the concept that electronegative polymers induce catalytic dysfunction of FXIa through binding to electropositive heparin binding sites located on the apple and catalytic domains [48,49].

To investigate the mechanism underlying the inhibitory effect of HSA-AGE on intrinsic coagulation, mixing studies were performed using factor-deficient plasmas. PTT-a–triggered clotting in FIX-deficient plasma was not affected by HSA-AGE compared with baseline FIX-deficient plasma. However, mixing FIX-deficient plasma with 5% PNP restored the HSA-AGE prolongation of clotting times, suggesting that HSA-AGE is additionally capable of inhibiting at the axis of FIXa. We pursued this finding and exposed FIX-deficient plasma to exogenous FIXa or FXa before adding HSA-AGE. Therefore, we confirmed that HSA-AGE inhibited clotting when FIXa was supplemented under these conditions but did not interfere with clotting when exogenous FXa was employed in the presence of PTT-a-triggered coagulation. In purified chromogenic assays, HSA-AGE inhibited FIXa-dependent FX activation; analysis of the kinetics of these reactions suggested that HSA-AGE was a competitive inhibitor of FX activation by FIXa.

There are limitations to the current study. The purified FIX-dependent FXa generation chromogenic assays were performed without cofactors FVIIIa or FVa, but we believe these cofactors may not be so critical because we demonstrate the anticoagulant effect of HSA-AGE in normal plasma. Therefore, we believe that the effects of FVIIIa or FVa may not be critical to the inhibition mechanism exerted by HSA-AGE to FIXa-dependent FX activation. Future studies should specifically address whether FIX or FX binding to misfolded protein was responsible for the phenotype of bleeding disorders previously reported in systemic amyloidosis [50].

The HSA-AGE inhibition toward the intrinsic pathway was not only limited to static PTT-a–triggered plasma assays because platelet-mediated fibrin formation using an *in vitro* flow-based model but was also dose-dependently prolonged. Therefore, providing direct evidence that the presence of procoagulant platelets does not override the inhibitory effect of HSA-AGE toward fibrin formation.

FXII is a protein that has critical roles in disease pathology, such as the acute sterile inflammation seen in hereditary angioedema and sepsis, but also chronic conditions, such as Alzheimer disease, systemic amyloidosis, and thrombosis [5,7,9,10]. The lack of an adverse phenotype with FXII deficiency makes FXII and its substrates attractive therapeutic targets. The present study identifies that HSA-AGE activates FXII-dependent PKa generation without inducing an accompanying increase in intrinsic coagulation due to its inhibitory effect on FIXa activation of FX, a novel mechanism described herein. These investigations add for the first time, mechanistic insight as to how misfolded proteins can trigger FXII-dependent PKa generation without any associated intrinsic coagulation. Further work is needed to investigate whether other physiologically relevant misfolded proteins can interact in a similar manner with coagulation system components.

## Supplementary Material

Supplement

## Essentials

- Advanced glycation end products (AGEs) are associated with hyperglycemia in diabetes mellitus.
- Human serum albumin (HSA)-AGE triggers factor XII activation of prekallikrein (PK).
- HSA-AGE inhibits intrinsic coagulation and triggers inflammation via FXII-induced activation of PK.
- HSA-AGE may trigger inflammatory rather than procoagulant responses by activating FXII/PK in diabetes mellitus.
