# Supplementary material for: Glycated albumin modulates the contact system with implications for the kallikrein-kinin and intrinsic coagulation systems
Source: J Thromb Haemost. Author manuscript; Available in PMC 2023 Jun 6. (PMC10242648; doi:10.1016/j.jtha.2022.12.015)
Supplement: Supplement [file NIHMS1899304-supplement-Supplement.docx]

## Supplemental methods

**Reagents.** FXIIa, PKa, PK, HK, and CTI were purchased from Enzyme Research Laboratories, FIX, FIXa, FX, FXa, FXI and FXIa were purchased from Haematologic Industries (VT, USA). Primary antibodies goat polyclonal anti-human FXII antibody were purchased from Enzyme research laboratories, while rabbit monoclonal anti-human HK was purchased from Abcam (Cambridge, UK), sheep polyclonal prekallikrein antibody was obtained from Affinity Biologicals, and goat polyclonal anti-human transferrin was purchased from Bio-Rad Antibodies (Hertfordshire, UK). Secondary antibodies HRP-conjugated goat polyclonal anti-rabbit was purchased from Agilent Technologies (Cheadle, UK), HRP-conjugated mouse polyclonal anti-goat was purchased from Insight Biotechnology (Wembley, UK) and HRP-conjugated donkey anti-sheep IgG was from Abcam. Plasmas with congenital deficiencies for FXII, PK, FXI, or FIX was purchased from George King Bio-medical (KS, USA). Chromogenic substrates S2288 and S2302 were purchased from Quadratech Diagnostics Limited (East Sussex, UK). PBS buffer tablets, D-glucose-6-phosphate disodium salt hydrate (G6P), NaN_3_, 4-(2-hydroxyethyl)-1-piperazineethanesulfonic acid [HEPES], NaCl, CaCl_2_, ZnCl_2_, MgCl_2,_ Kaolin, human serum albumin, and bovine serum albumin were purchased from Sigma-Aldrich (Dorset, UK). Thrombin calibrator, FluCat kit, PTT-automate 5 silica-activator (PTT-a), Technoplastin-HIS, STA-Reptilase, and Owen Koller diluent were purchased from Stago (Theale, UK). Synthetic phospholipids DOPC, DOPS, and DOPE were purchased from Avanti Polar Lipids (AL, USA).

**Blood collection and plasma preparation***.* Blood samples were drawn from healthy donors after informed consent via venipuncture into BD plus plastic vacutainers™ containing a 10% volume of 0.109 M sodium citrate. The first 1.8 mL sodium citrate vacutainer following venipuncture was discarded to avoid TF contamination. Ethical approval was received from the University of Leeds Medical School ethical committee (HSLTLM/12/045). Platelet-poor plasma was obtained by centrifugation of blood-filled vacutainers at 2000 RCF for 5 minutes, then ¾ of the plasma supernatant was then carefully removed and further centrifuged at 10,000 RCF for 15 minutes to pellet any residual cellular material. The supernatant was then filtered using a 0.22 µm syringe filter. Pooled normal plasma (PNP) was prepared by pooling platelet-poor plasma from 27 healthy donors (16 males, 11 females, mean age of 31.5 ± 1.4 years and BMI 24.6 ± 0.6 Kg/m^2^ [data expressed as mean ± SEM]).

**Human Studies***.* All clinical sample studies were conducted following the principles of the Declaration of Helsinki. All participants provided written informed consent. The protocol, amendments, and informed consent forms were approved by the Institutional Review Board (Case 12Z05, IRB # 09-90-195) of University Hospitals Cleveland Medical Center and the Louis Stokes Cleveland Veterans Administration Medical Center (CY18-051). Eligible non-diabetic subjects were 18 years or older, male and female; were without a diagnosis of an acute illness in the past 4 weeks; had no prior diagnosis of diabetes mellitus (DM) determined by review of their health records and defined as fasting blood glucose level < 100 mg/dl, random blood glucose level <200 mg/dL and/or glycated hemoglobin A1C (Hgb A1C) ≤5.6%, measured by turbidimetric inhibition immunoassay Tina-quant^®^ HbA1c Gen. 3 assay (Roche) at the Louis Stokes Cleveland VA Medical Center central laboratory. Diabetic participants were similarly 18 years or older; male or female; had previously been diagnosed with DM type I or II, defined as fasting blood glucose within 3 months from accrual of ≥126 mg/dL, random blood glucose level greater than or equal to 200 mg/dl, and Hgb A1C ≥6.5% repeated at least twice for confirmation, according to the American Diabetes Association guidelines; who were pharmacologically treated for their DM. We also accrued 2 individuals with impaired glucose tolerance (IGT), defined as fasting glucose level within 3 months of accrual 100–125 mg/dL or Hgb A1C 5.7-6.4%. For all participants, whole blood was drawn by venipuncture into sodium citrate tubes (ratio: 1-part anticoagulant to 9 parts whole blood) and all tubes were deidentified before transporting to the laboratory. To obtain platelet-poor plasma (PPP), whole blood was centrifuged sequentially at 3,000g for 30 min, followed by 11,000g for 10 min at room temperature. PPP supernatants were divided into 0.25 ml aliquots, transferred into clean polypropylene tubes, and stored at -80°C. Detection of AGE-specific peptides in plasma were detected as described in supplemental methods. All research laboratory members performing measurements were blinded as to the glycemic control of each sample.

**Immunoblotting of patient samples*.*** Plasma samples were diluted (1:30) and mixed with 2X Laemmli buffer which was reduced with 5% β-mercaptoethanol. Equal amounts of total protein were loaded, resolved, and transferred onto nitrocellulose. The membranes were incubated with purified primary polyclonal FXII antibody (Affinity Biologicals, 1:1000 dilution) and transferrin (Abcam, 1:10,000 dilution) for 2 h. For prekallikrein and HK detection, samples were processed as above but incubated overnight with primary polyclonal prekallikrein (Affinity Biologicals, 1:1000 dilution) and HK rabbit monoclonal (Abcam, 1:10,000 dilution) antibodies, respectively, in that order. Blots were stripped between antibody incubations and developed using enhanced chemiluminescent substrate (Pierce). Primary antibodies for FXII were detected with a horseradish peroxidase (HRP)–conjugated anti–goat immunoglobulin G (Santa Cruz, 1:5000), PK was detected with horseradish peroxidase–conjugated anti–sheep immunoglobulin G (Abcam, 1:5000) for 1 h and HK with secondary HRP-conjugated goat polyclonal anti-rabbit antibody. Where indicated, non-reduced plasma samples were probed for HK and similarly detected as indicated above. The blots were scanned using Scion Image (v4.0) software. Protein levels were quantified using densitometric analysis with ImageJ (NIH). To quantify FXII and HK cleavage, the lane density for FXIIa heavy chain (52 kDa), HK light chain (56 kDa), and HK light chain fragment (45 kDa) was normalized to the band density of zymogen FXII (~78 kDa) and intact HK (120 kDa), respectively and presented as % relative densitometry units (RDU). To quantify PK antigen, the lane density for PK (85 kDa) was normalized to the band density of transferrin (80 kDa) and presented as % RDU.

**Immunoblotting for FXII and HK activation *in vitro.*** Reactions containing PNP were conducted in polypropylene tubes at 37ºC. PNP was diluted (1:5) in HBS buffer (10 mM HEPES, 10 µM ZnCl_2_, 50 mM NaCl, 1% PEG-8000, pH 7.4), and incubated for 3 hours with either PTT-a (20% v/v) or HSA-AGE (250 µg.mL^-1^). Reactions were quenched by diluting the reaction mixture (1:40) in 1M NaCl. Samples were then separated by SDS-PAGE and transferred onto PDVF membranes. Blots of reduced PNP were probed with primary antibody followed by secondary antibody HRP-conjugated mouse polyclonal anti-goat. Blots of Blots of non-reduced PNP were probed with primary antibody rabbit polyclonal anti-human HK followed by secondary antibody HRP-conjugated goat polyclonal anti-rabbit. For loading controls, blots were stripped and probed with primary antibody goat polyclonal anti-human transferrin followed by secondary antibody HRP-conjugated mouse polyclonal anti-goat.

**Detection of AGE-specific peptides in plasma.** PPP was diluted in phosphate buffered saline (10% plasma v/v) and fluorescence intensity of plasma was measured at 440 nM after excitation at 345 nm using a Promega GloMax® Discover microplate reader. A calibration curve of rhodamine 6G fluorescence was used to normalize readings. AGE-specific fluorescence was previously characterized by Yanagisawa et al [1].

**Albumin glycation and preparation of surfaces for contact activation.** Advanced glycation end products of bovine serum albumin (BSA-AGE) was prepared by incubating 100 mg.mL^-1^ BSA (Albumin from bovine serum A4737, Sigma-Aldrich) for 23 weeks in the dark at 37°C in PBS buffer (137 mM sodium chloride, 2.7 mM potassium chloride, 10 mM disodium hydrogen phosphate, 1.8 mM potassium di-hydrogen phosphate, pH 7.4), 1 M D-glucose-6-phosphate disodium salt hydrate (G6P) and 0.05% (w/v) NaN_3_. Control BSA was prepared in the same way as BSA-AGE but G6P was omitted, and HSA prepared on the day of each experiment. Following glycation after 23 weeks, solutions of BSA-AGE were dialyzed against HBS for 24 hours at 4ºC, with buffer changes at 16, and 20 hours. AGE-specific autofluorescence was measured using excitation 485nm, emission 530nm using a PowerWave HT Microplate Spectrophotometer (BioTek, UK). We also prepared Advanced glycation end products of human serum albumin (HSA-AGE), prepared in the same way as BSA-AGE, however, we incubated HSA for a longer period of 35 weeks with G6P. Concentrations of HSA-AGE/BSA-AGE were determined using standard BCA assays (sigma). 10 μg of each HSA preparation was loaded onto a NativePAGE Novex 3-12% Bis-Tris gel following the NativePAGE workflow as described by the manufacturer (Life Technologies, Warrington, UK). Heat denatured BSA, and HSA (dBSA/dHSA) was prepared by heating 10 mg.mL^-1^ BSA/HSA in HBS buffer to 85ºC for 10 minutes. Kaolin (K7375, Sigma-Aldrich) was made to stock concentrations of 1 mg.mL^-1^ in HBS buffer for each experiment and left on a roller for 30 minutes to achieve a fine particle suspension. PTT-a was prepared according to the manufacturer’s instructions.

**Phospholipid preparation*.*** Phospholipids (Avanti Polar Lipids, AL, USA) were prepared and extruded as described in [2]. Phospholipids were supplied in chloroform and then dried under a stream of nitrogen gas. Dried phospholipids were then resuspended in HBS buffer (10 mM HEPES, 150 mM NaCl, pH 7.4). Micelles were prepared by mixing 20% di-oleic phosphatidylethanolamine (DOPE), 20% di-oleic phosphatidylserine (DOPS), and 60% di-oleic phosphatidylcholine (DOPC) and extruded through a membrane with a pore size of 1μm.

**Thioflavin-T and Congo red fluorescence assay.** Assays were conducted in Nunc Microwell black 96-well optical bottom plates (Thermo-Fisher). 5 mM stock concentrations of Thioflavin-T (ThT) and Congo red (CR) were prepared by mixing either with DMSO and were left to fully solvate with continuous stirring, for 30 minutes, in the dark. Solutions of misfolded proteins were used at final concentrations of 100 µg.mL-1 and were incubated with either 10 µM ThT or 20 µM CR (final concentrations) and incubated at room temperature for 10 minutes in the dark. All assays had a final volume of 100 µL and were conducted in HBS buffer (10 mM HEPES, 150 mM NaCl, pH 7.4). Excitation wavelengths of 445nm and emission 482 nm was used to measure ThT fluorescence and excitation wavelengths of 550nm and emission 590nm was used to measure CR fluorescence. Data were background subtracted to compensate for any fluorescence exerted by the protein and expressed as a fold increase of fluorescence compared to solutions of ThT or CR in HBS.

**Chromogenic assays.** All chromogenic assays were conducted in 96-well clear microtiter plates blocked with 1% (w/v) PEG-20000. Cleavage of chromogenic substrates and appearance of pNA was monitored by a PowerWave HT Microplate Spectrophotometer (BioTek) at 37˚C at 405 nm every 12 seconds for 120 minutes unless otherwise stated. All chromogenic assays had a final reaction mixture of 100μL. All substrates were solvated in HBS unless otherwise stated. Rates of zymogen activation from continuous chromogenic assays in purified reactions or plasma were determined by linear regression analysis of absorbance vs. time squared plots for early time points of the reaction. A 120 s delay was included for the time between reaction mixing and the first reading. The resultant slopes (OD/s^2^) provided the zymogen activation rate and using the Shiny app (version 0.62)[3], the reaction rate was calculated in pM/s*.*

**Circular dichroism.** Spectra were obtained using a Chrirascan Plus (Applied Photophysics) spectropolarimeter; with a 6-cell charger, Peltier temperature control, stopped-flow capabilities, and automated temperature ramping. Far UV circular dichroism (CD) ellipticity was measured in a quartz cuvette with a path length of 1 mm (Hellma), between 180 nm and 260 nm in 1 nm increments and 1-second sample time. Near UV ellipticity was measured in a quartz cuvette with a path length of 1 cm (Hellma), between 250 nm and 320 nm within 1 nm increments and 1-second sample time. Protein samples used for Far UV CD were used at a concentration of 0.25 mg.mL^-1^, and for Near UV CD, 1 mg.mL^-1^. Protein samples for all experiments were extensively dialyzed against 50 mM NaH_2_PO_4_ pH 7.4. Spectra from Far UV CD were analyzed with the online server DichroWeb[4, 5] using the SELCON3 algorithm and reference data set 1.

**Data analysis***.* All data were analyzed with GraphPad Prism 9.0 for MacOS which was used to draw tables, perform statistical analysis, and construct graphical charts. Data sets containing two-groups were analyzed by student t-test. Data sets containing more than two groups were analyzed by a two-tailed Mann-Whitney test for non-parametric data, or one-way ANOVA with Sidak’s multiple comparisons test for parametric data. Groups were considered significant statistically different when p<0.05.

**Supplemental data**

|  | Non-diabetic  n = 10 | IGT  n=2 | Diabetic  n = 10 |
| --- | --- | --- | --- |
| Age - yr. | **61.6 ± 8.7** | **68.5 ± 3.5** | **66.6 ± 9.2** |
| Sex – no. (%)  Male  Female | 5 (50%)  5 (50%) | 2 (100%) | 8 (80%)  2 (20%) |
| Race – no. (%)  Caucasian  African American  Other | 5 (50%)  5 (50%) | 2 (100%) | 7 (70%)  2 (20%)  1 (10%) |
| Body-mass index – kg/m^2^ | 25.1 ± 3.1 | 33 ± 5.6 | 30.9 ± 5.3 |
| Estimated Glomerular filtration rate – no. (%)  ≥ 80 ml/min  50 – 80 ml/min  30 – 50 ml/min | 7 (70%)  2 (20%)  1 (10%) | 1 (50%)  1 (50%)  0 | 3 (30%)  7 (70%)  0 |
| Liver function –  Bilirubin, total (mg/dL)  Aspartate aminotransferase (U/liter)  Alanine aminotransferase (U/liter)  Alkaline phosphatase (U/liter) | 0.5 ± 0.1  27 ± 11  34 ± 13  83 ± 26 | 0.5 ± 0.3  24 ± 3  31 ± 7  76 ± 12 | 0.5 ± 0.2  23 ± 9  32 ± 13  78 ± 20 |
| Complete Blood Counts  WBC (K/cmm)  Hgb (g/dL)  Platelets (K/cmm) | 6.8 ± 3.5  14.5 ± 2.3  227 ± 89 | 6.75 ± 2.7  15 ± 1  175 ± 12 | 8.8 ± 4.6  14.2 ± 2.4  258 ± 90 |
| Coagulation Tests  Prothrombin Time (s)  Activated Partial Thromboplastin Time (s) | 10 ± 1  32.1 ± 3 | 11 ± 0.5  34.5 ± 2 | 10.8 ± 2  28.4 ± 3.5^#^ |
| DM Type I (%)  DM Type II (%)  Hemoglobin A1C – mean % | 5.5 | 6.2 | 2 (20%)  8 (80%)  7.58 |
| Comorbidities – incidence (%)  Hypertension  Hyperlipidemia  Coronary Artery Disease*  Arterial thrombotic events^§^  Venous thromboembolic events^ʭ^ | 50%  30%  10%  0%  0% | 100%  50%  50%  10%  0% | 80%  60%  30%  20%  20% |

**Supplemental Table 1. Demographics and clinical characteristics of patients**. Data presented as mean ± SD. Comparisons within groups for liver function, complete blood counts, and coagulation tests were performed using two-way ANOVA. Groups that showed statistical differences were then subject to Dunnett’s post-hoc multiple comparisons test when *p*<0.05, rows were compared to the non-diabetic control group. Significance *p*<0.05(^#^). *Coronary artery disease: documented in patients’ medical record and identified by prior acute myocardial infarction, positive stress test, evidence of coronary stenosis by cardiac catheterization, or presence of cardiac stents. ^§^Arterial thrombotic events: defined as de novo or embolic thrombosis in an artery. ^ʭ^Venous thromboembolic events: defined as in situ or embolic thrombosis in a deep vein. Superficial vein thrombosis was excluded from this group.

**Supplemental Figure 1. Amidolytic activity towards S2302 in plasma following PTT-a triggered contact activation is dependent on PKa activity.** (A) PTT-a initiated S2302 cleavage in PNP or plasma from donors with a congenital deficiency for FXII, PK, FXI, FIX, FX, or FVII. (B) Rate of S2302 cleavage in pooled normal plasma (PNP) in the presence CTI or SBTI (CTI; 0.156 – 10 μM, SBTI; 0.156 - 5 μM). Inhibitors were added to PNP before the addition of PTT-a (labelled before PTT-a) or following a five-minute incubation period for PTT-a to generate PKa (labelled as “after PTT-a”). Data is presented as means ± SD, means are constructed of triplicate readings.

**Supplemental Figure 2. BSA-AGE has AGE-specific autofluorescence, forms multimers and has different structural features compared to native albumin.** (A) BSA was glycated with glucose-6-phosphate (G6P) for 23 weeks at 37ºC, and as a control, G6P was omitted. Autofluorescence was detected in the BSA-AGE solution, indicative of the formation of advanced glycation end products. (B) A transition of albumin secondary structure was indicated by the increase in fluorescence of solutions made of ThT and albumins. (C) Incubation of albumins with the fibrillar amyloid-specific dye CR (D) Non-reducing SDS-PAGE gel of fresh BSA, control BSA (23 weeks), and BSA-AGE. (E) Far UV CD spectra (left) demonstrates BSA-AGE and dBSA secondary structural deviations compared to fresh albumin. Near UV CD spectra (right) shows changes in protein tertiary structure. (F) Structure fractions were determined from the spectra obtained in Far UV CD in panel E. *where significantly different to the BSA control. *p<0.05, **p<0.01, ***p<0.001, ****p<0.0001. Statistical comparisons were made using two-way ANOVA with Dunnett’s multiple comparisons test.

**Supplemental Figure 3. BSA-AGE generates FXII dependent PK activity *in vitro.*** FXII dependent PKa generation shown by a continuous chromogenic assay. (A) FXII dependent PKa activity was triggered by BSA-AGE but not without a surface. (B) Concentration of up to 100 μg.mL^-1^ (full concentration ranges not shown) of fresh BSA or dBSA did not induce FXII dependent PKa generation. As a positive control, PTT-a was added to induce PKa activity. (C) Titrations of BSA-AGE dose-dependently triggered purified FXII amidolytic activity towards S2302. Data shown as rate of S2302 cleavage (ΔmOD.min^-1^). (D) PKa-like activity chromogenic activity assay performed with pooled normal plasma (PNP) or plasmas with deficiencies for FXII, PK, FXI, FIX, FX, or FVII. PKa was generated using 50 μg.mL^-1^ BSA-AGE in each plasma. PTT-a was used as a positive control, and HBS was used as the negative control in PNP. (E) 10mM ThT was incubated with 100 μg.mL^-1^ BSA-AGE, dBSA, or native BSA and visualized by fluorescence microscopy.

**Supplemental Figure 4. Purified FXII dependent PKa generation chromogenic assay. PKa generation is dependent on the presence of FXII, HK, and an anionic surface.** 2 nM FXII was incubated with 20 nM PK, 20 nM HK, PTT-**a,** or HSA-AGE for 40 minutes prior to adding 2 mM S2302. The change in absorbance (OD405nm) was measured every 12s for a total of 120s. (A) A S-2302 proteolysis was absent in reactions of FXII and PK, and a low rate of proteolysis was seen in reactions containing FXII, PK, and HK in the absence of a surface. S-2302 proteolysis was not seen in control reactions containing only 2 nM FXIIa. (B) PKa generation is increased with PTT-a in reactions containing either FXII + PK, and FXII + PK + HK. Proteolysis of S2302 was not seen in any other reaction. Data points are mean ± SD of assays performed in triplicate; error bars may not visible.

**Supplemental Figure 5. Purified FXII dependent FXIa generation chromogenic assay.** FXIa generation is dependent on the presence of FXII, HK, and an anionic surface. 2 nM FXII, 30 nM FXI, 30 nM HK, and PTT-a were incubated for 3 hours prior to the addition of 2 mM S2288. The change in absorbance (OD405nm) was measured every 12s for a total of 600s. (A) FXIa was not generated in the absence of either FXII, HK, or PTT-a. 2 nM FXIIa alone was unable to cleave S-2288. (B) FXIa generation is increased with PTT-a in reactions containing either FXII + FXI, and FXII + FXI + HK. Proteolysis of S2288 was not seen in any other reaction. Data points are mean ± SD of assays performed in triplicate.

**Supplemental Figure 6. Characterization of *in vitro* generated HSA-AGE.** (A) HSA was denatured via glycation or thermal treatment. A transition of HSA secondary structure was indicated by the increase in fluorescence of solutions of ThT. Data is presented as means ± SD, means are constructed of triplicate readings. (B) Incubation of denatured HSA with CR. (C) NativePAGE performed with fresh HSA, control HSA (incubated for 35 weeks in PBS), and HSA-AGE which shows the native molecular weight of the proteins in suspension. A total of 10 μg of each protein was loaded into each gel lane. *p<0.05, **p<0.01, ***p<0.001, ****p<0.0001. Statistical comparisons were made using two-way ANOVA with Dunnett’s multiple comparisons test.

**Supplemental Figure 7. aPTT of PNP, FXII-deficient, PK-deficient, FXI-deficient, and FIX-deficient plasmas used for clotting experiments.** Assay limit was set to the standard 300s. Data shown as mean ± SD of assays performed in triplicate.

**Supplemental Figure 8. HSA-AGE does not inhibit FXa or FIXa amidolytic activity towards S2765**. Purified FXa (3 nM) or FIXa (300nM) was incubated with titrations of HSA-AGE 0.78 - 50 μg.mL^-1^ and S2765. 700 μM S2765 was added to reactions with FXa, and 3 mM S2765 was added to reaction containing FIXa. The initial rates of S2765 proteolysis were measured and expressed relative to control solutions (%). Data shown are mean ± SD of experiments performed in triplicate.

**Supplemental fig 9. Platelet and fibrin surface area coverage is dependent on immobilized collagen using an *in vitro* flow assay.** (A) Fibrin formation and platelet area coverage under flow conditions in human blood is dependent on the presence of collagen. Data presented as mean % area coverage ± SEM of 3 donors. (B) AUC of curves from panel A. Data presented as mean AUC ± SEM of triplicate data collected from 3 donors. *p<0.05, **p<0.001, ***p<0.0005, ****p<0.0001. ns, no statistical difference.

**Supplemental Figure 1. Amidolytic activity towards S2302 in plasma following PTT-a triggered contact activation is dependent on PKa activity.** (A) PTT-a initiated S2302 cleavage in PNP or plasma from donors with a congenital deficiency for FXII, PK, FXI, FIX, FX, or FVII. (B) Rate of S2302 cleavage in pooled normal plasma (PNP) in the presence CTI or SBTI (CTI; 0.156 – 10 μM, SBTI; 0.156 - 5 μM). Inhibitors were added to PNP before the addition of PTT-a (labelled before PTT-a) or following a five-minute incubation period for PTT-a to generate PKa (labelled as “after PTT-a”). Data is presented as means ± SD, means are constructed of triplicate readings.


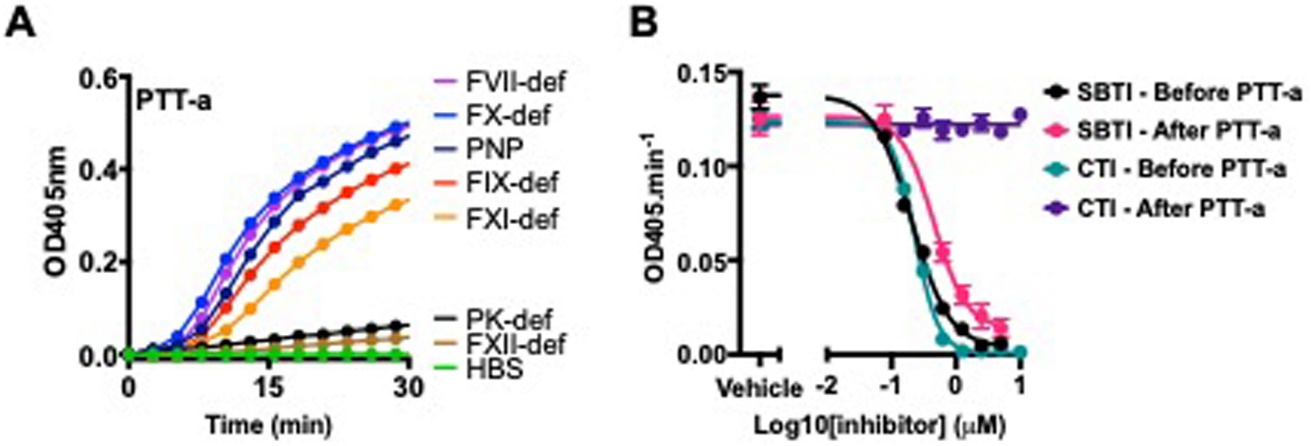


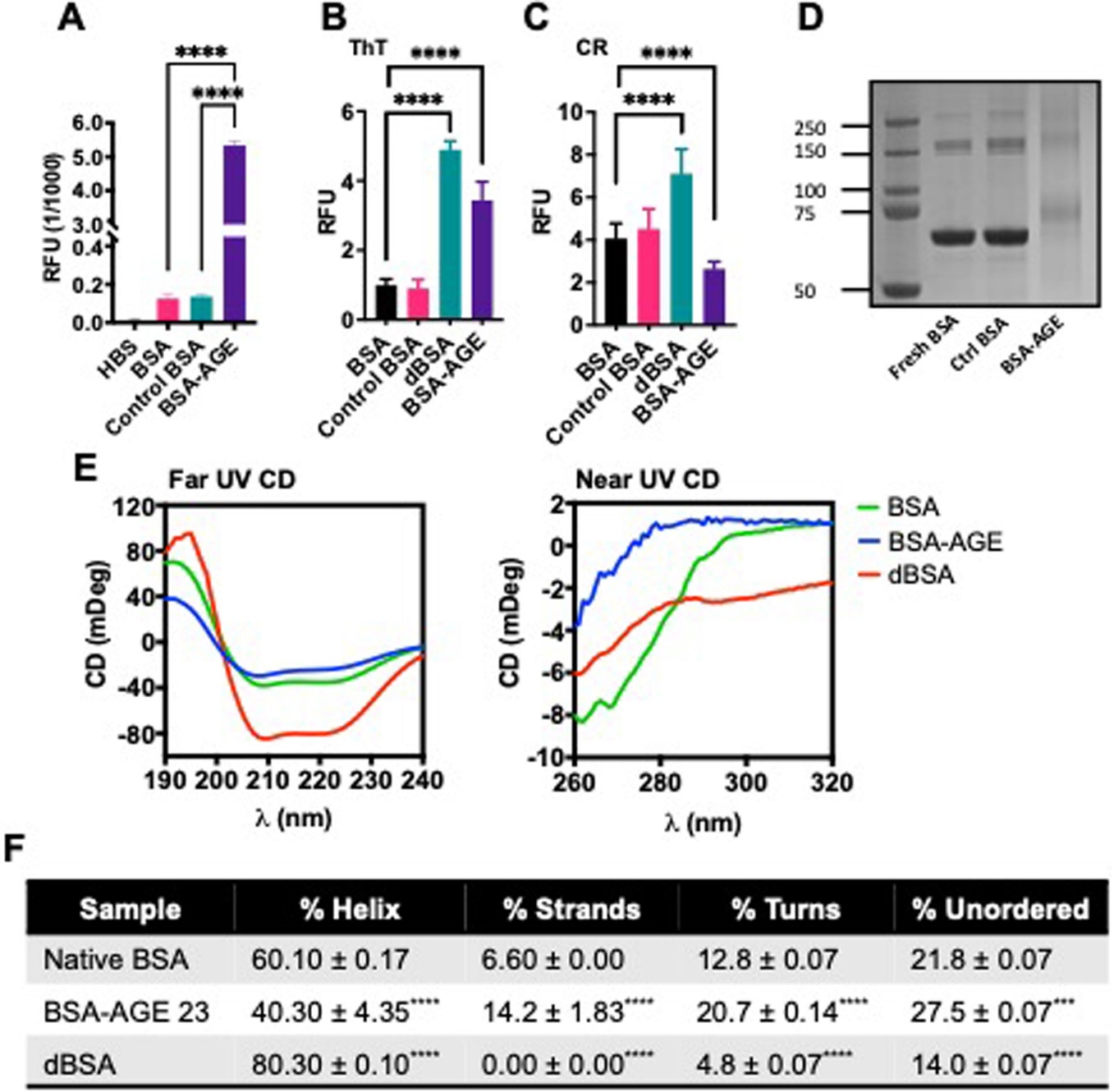
**Supplemental Figure 2. BSA-AGE has AGE-specific autofluorescence, forms multimers and has different structural features compared to native albumin.** (A) BSA was glycated with glucose-6-phosphate (G6P) for 23 weeks at 37ºC, and as a control, G6P was omitted. Autofluorescence was detected in the BSA-AGE solution, indicative of the formation of advanced glycation end products. (B) A transition of albumin secondary structure was indicated by the increase in fluorescence of solutions made of ThT and albumins. (C) Incubation of albumins with the fibrillar amyloid-specific dye CR (D) Non-reducing SDS-PAGE gel of fresh BSA, control BSA (23 weeks), and BSA-AGE. (E) Far UV CD spectra (left) demonstrates BSA-AGE and dBSA secondary structural deviations compared to fresh albumin. Near UV CD spectra (right) shows changes in protein tertiary structure. (F) Structure fractions were determined from the spectra obtained in Far UV CD in panel E. *where significantly different to the BSA control. *p<0.05, **p<0.01, ***p<0.001, ****p<0.0001. Statistical comparisons were made using two-way ANOVA with Dunnett’s multiple comparisons test.

**Supplemental Figure 3. BSA-AGE generates FXII dependent PK activity *in vitro.*** FXII dependent PKa generation shown by a continuous chromogenic assay. (A) FXII dependent PKa activity was triggered by BSA-AGE but not without a surface. (B) Concentration of up to 100 μg.mL^-1^ (full concentration ranges not shown) of fresh BSA or dBSA did not induce FXII dependent PKa generation. As a positive control, PTT-a was added to induce PKa activity. (C) Titrations of BSA-AGE dose-dependently triggered purified FXII amidolytic activity towards S2302. Data shown as rate of S2302 cleavage (ΔmOD.min^-1^). (D) PKa-like activity chromogenic activity assay performed with pooled normal plasma (PNP) or plasmas with deficiencies for FXII, PK, FXI, FIX, FX, or FVII. PKa was generated using 50 μg.mL^-1^ BSA-AGE in each plasma. PTT-a was used as a positive control, and HBS was used as the negative control in PNP. (E) 10mM ThT was incubated with 100 μg.mL^-1^ BSA-AGE, dBSA, or native BSA and visualized by fluorescence microscopy.


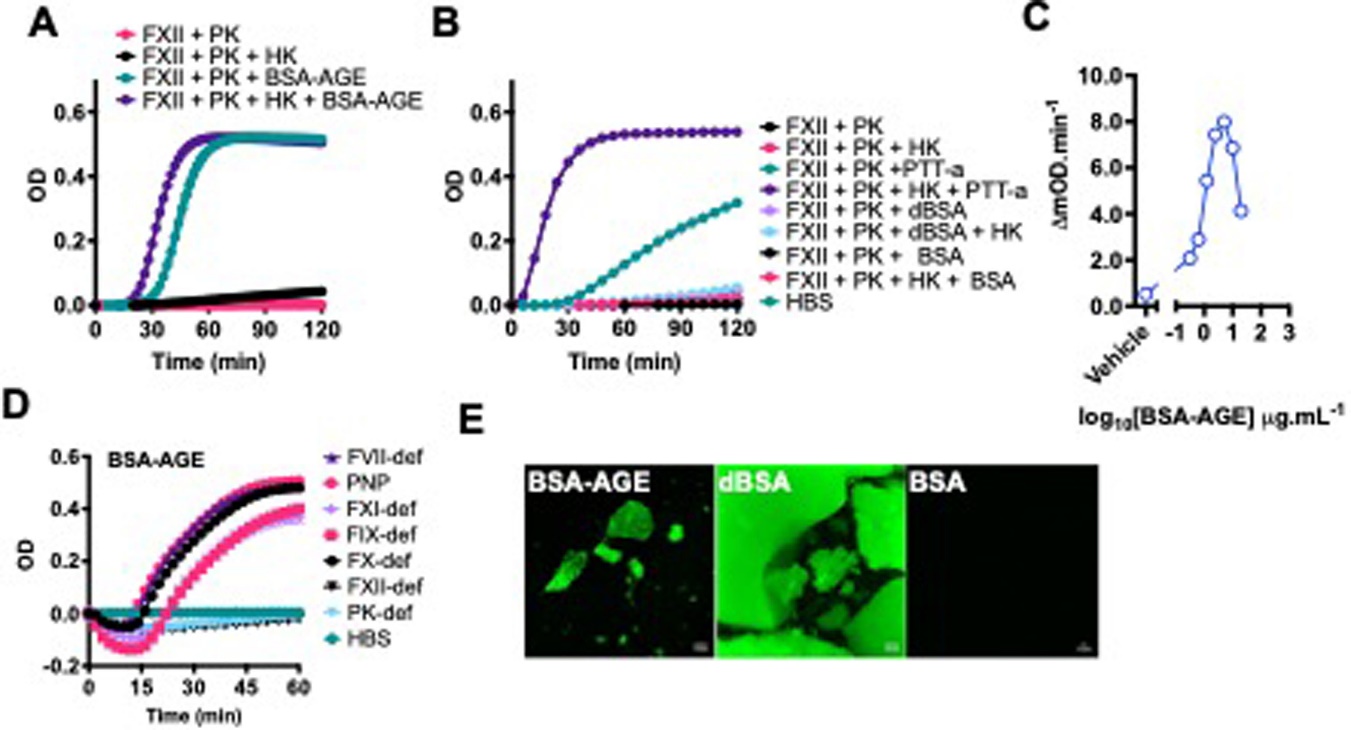


**Supplemental Figure 4. Purified FXII dependent PKa generation chromogenic assay. PKa generation is dependent on the presence of FXII, HK, and an anionic surface.** 2 nM FXII was incubated with 20 nM PK, 20 nM HK, PTT-**a,** or HSA-AGE for 40 minutes prior to adding 2 mM S2302. The change in absorbance (OD405nm) was measured every 12s for a total of 120s. (A) A S-2302 proteolysis was absent in reactions of FXII and PK, and a low rate of proteolysis was seen in reactions containing FXII, PK, and HK in the absence of a surface. S-2302 proteolysis was not seen in control reactions containing only 2 nM FXIIa. (B) PKa generation is increased with PTT-a in reactions containing either FXII + PK, and FXII + PK + HK. Proteolysis of S2302 was not seen in any other reaction. Data points are mean ± SD of assays performed in triplicate; error bars may not visible.
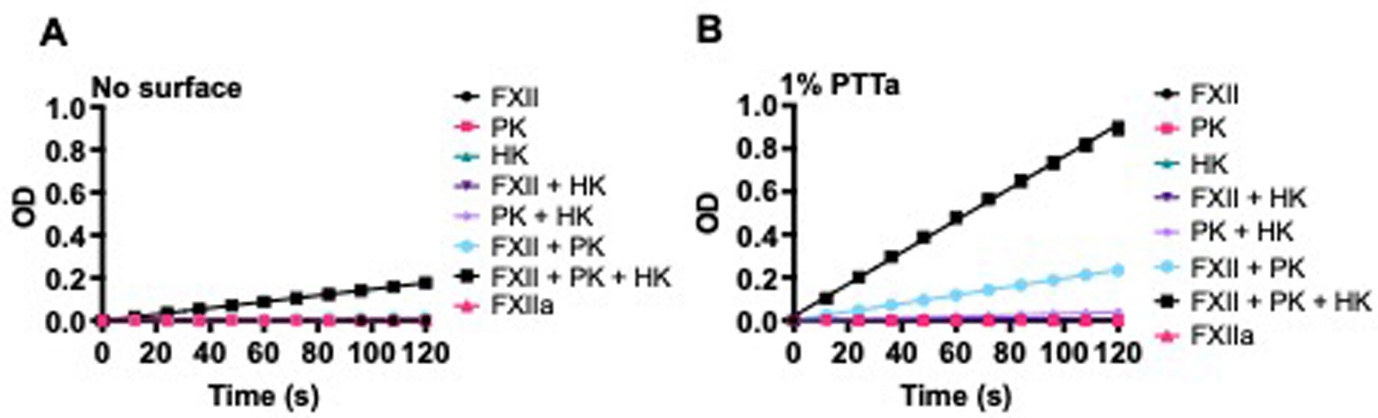


**Supplemental Figure 5. Purified FXII dependent FXIa generation chromogenic assay.** FXIa generation is dependent on the presence of FXII, HK, and an anionic surface. 2 nM FXII, 30 nM FXI, 30 nM HK, and PTT-a were incubated for 3 hours prior to the addition of 2 mM S2288. The change in absorbance (OD405nm) was measured every 12s for a total of 600s. (A) FXIa was not generated in the absence of either FXII, HK, or PTT-a. 2 nM FXIIa alone was unable to cleave S-2288. (B) FXIa generation is increased with PTT-a in reactions containing either FXII + FXI, and FXII + FXI + HK. Proteolysis of S2288 was not seen in any other reaction. Data points are mean ± SD of assays performed in triplicate.


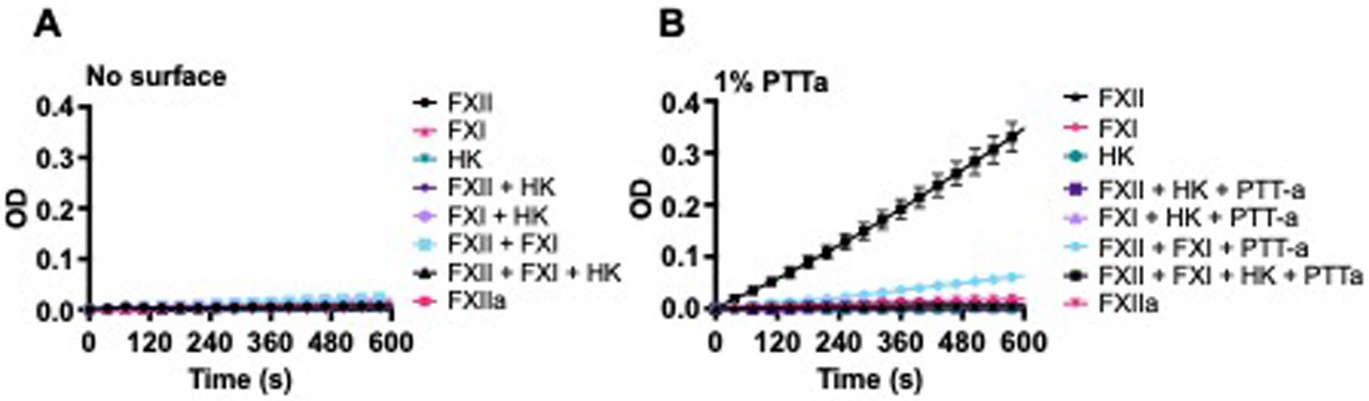


**Supplemental Figure 6. Characterization of *in vitro* generated HSA-AGE.** (A) HSA was denatured via glycation or thermal treatment. A transition of HSA secondary structure was indicated by the increase in fluorescence of solutions of ThT. Data is presented as means ± SD, means are constructed of triplicate readings. (B) Incubation of denatured HSA with CR. (C) NativePAGE performed with fresh HSA, control HSA (incubated for 35 weeks in PBS), and HSA-AGE which shows the native molecular weight of the proteins in suspension. A total of 10 μg of each protein was loaded into each gel lane. *p<0.05, **p<0.01, ***p<0.001, ****p<0.0001. Statistical comparisons were made using two-way ANOVA with Dunnett’s multiple comparisons test.


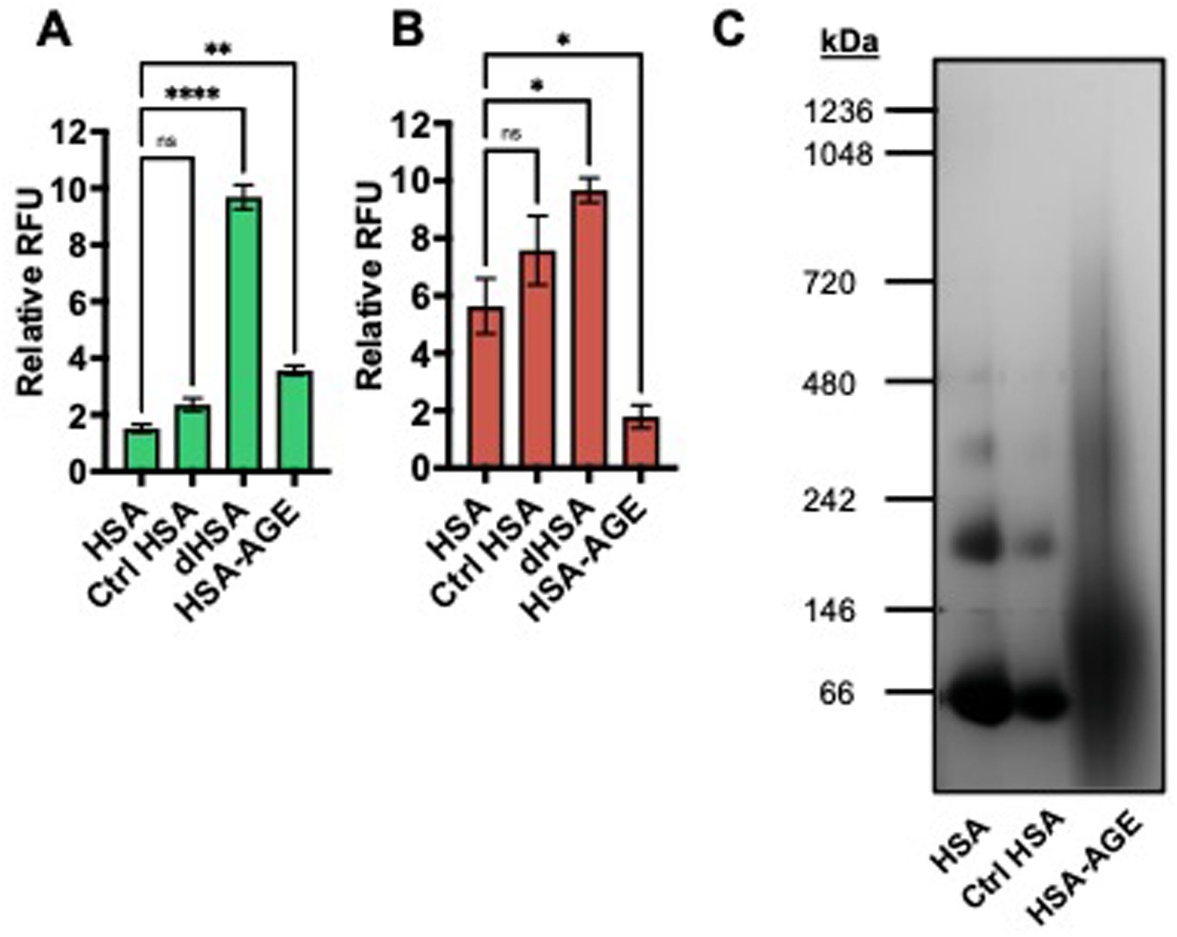


**Supplemental Figure 7. aPTT of PNP, FXII-deficient, PK-deficient, FXI-deficient, and FIX-deficient plasmas used for clotting experiments.** Assay limit was set to the standard 300s. Data shown as mean ± SD of assays performed in triplicate.


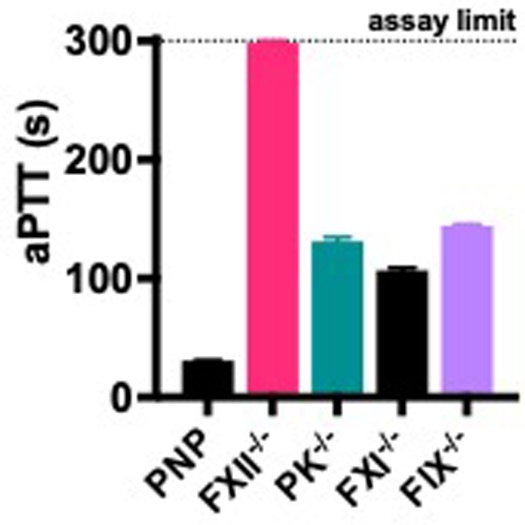


**Supplemental Figure 8. HSA-AGE does not inhibit FXa or FIXa amidolytic activity towards S2765**. Purified FXa (3 nM) or FIXa (300nM) was incubated with titrations of HSA-AGE 0.78 - 50 μg.mL^-1^ and S2765. 700 μM S2765 was added to reactions with FXa, and 3 mM S2765 was added to reaction containing FIXa. The initial rates of S2765 proteolysis were measured and expressed relative to control solutions (%). Data shown are mean ± SD of experiments performed in triplicate.


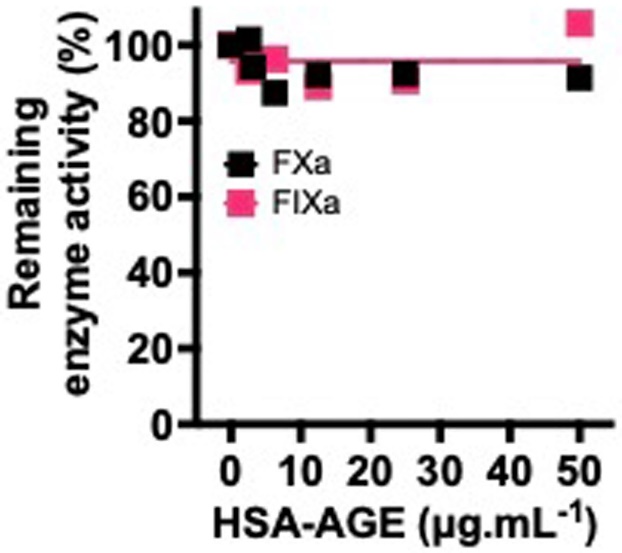


**Supplemental fig 9. Platelet and fibrin surface area coverage is dependent on immobilized collagen using an *in vitro* flow assay.** (A) Fibrin formation and platelet area coverage under flow conditions in human blood is dependent on the presence of collagen. Data presented as mean % area coverage ± SEM of 3 donors. (B) AUC of curves from panel A. Data presented as mean AUC ± SEM of triplicate data collected from 3 donors. *p<0.05, **p<0.001, ***p<0.0005, ****p<0.0001. ns, no statistical difference.


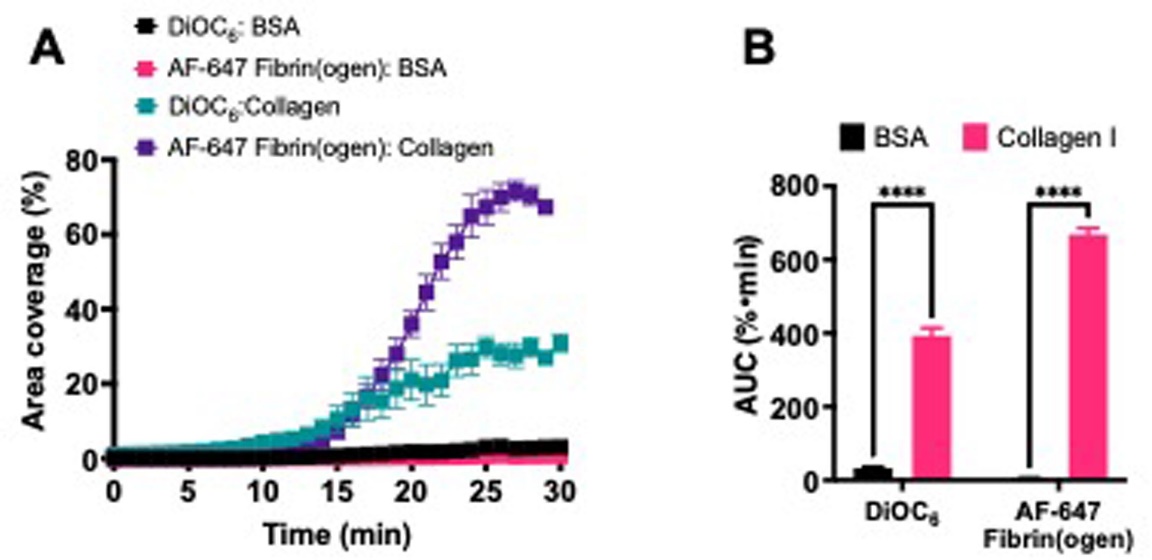


**References**

1. Yanagisawa, K., et al., Specific fluorescence assay for advanced glycation end products in blood and urine of diabetic patients. Metabolism, 1998. 47(11): p. 1348-53.

2. Ahnström, J., et al., Partial rescue of naturally occurring active site factor X variants through decreased inhibition by tissue factor pathway inhibitor and antithrombin. J Thromb Haemost, 2020. 18(1): p. 136-150.

3. Longstaff, C. and s.o. fibrinolysis, Development of Shiny app tools to simplify and standardize the analysis of hemostasis assay data: communication from the SSC of the ISTH. J Thromb Haemost, 2017. 15(5): p. 1044-1046.

4. Lobley, A., L. Whitmore, and B.A. Wallace, DICHROWEB: an interactive website for the analysis of protein secondary structure from circular dichroism spectra. Bioinformatics, 2002. 18(1): p. 211-2.

5. Whitmore, L. and B.A. Wallace, DICHROWEB, an online server for protein secondary structure analyses from circular dichroism spectroscopic data. Nucleic Acids Res, 2004. 32(Web Server issue): p. W668-73.
